# Recent Advances in Calixarene-Based Fluorescent Sensors for Biological Applications

**DOI:** 10.3390/s24227181

**Published:** 2024-11-08

**Authors:** Paula M. Marcos, Mário N. Berberan-Santos

**Affiliations:** 1 Centro de Química Estrutural, Institute of Molecular Sciences, Faculdade de Ciências, Universidade de Lisboa, Edifício C8, 1749-016 Lisboa, Portugal; 2 Faculdade de Farmácia, Universidade de Lisboa, Av. Prof. Gama Pinto, 1649-003 Lisboa, Portugal; 3 IBB-Institute for Bioengineering and Biosciences, Instituto Superior Técnico, Universidade de Lisboa, 1049-001 Lisboa, Portugal; berberan@tecnico.ulisboa.pt

**Keywords:** calixarenes, fluorescent sensors, detection mechanisms, ions and biomolecules, biological applications, cells and bioimaging, drug delivery, cancer therapy, nanomaterials

## Abstract

Due to their structural features, macrocyclic compounds such as calixarenes, conjugated with a variety of fluorophores have led to the development of fluorescent probes for numerous applications. This review covers the recent advances (from 2009 to date) made in calixarene-based fluorescent sensors and their biological applications. In addition to the fluorescence mechanisms used to signal the analyte binding, this article focuses mainly on the detection of biological relevant ions, on the selective sensing of biomolecules, such as amino acids, enzymes, drugs and other organic compounds, and on intracellular imaging. Calixarene-containing fluorescent nanoparticles and nanoaggregates for imaging and drug delivery are also described. Finally, this review presents some conclusions and future perspectives in this field.

## 1. Introduction

Supramolecular interactions are present in various metabolic functions in living organisms. Inspired by nature, researchers from various fields have developed numerous macrocyclic synthetic hosts for different applications in areas such as sensing, catalysis, nanomaterials and drug delivery.

The detection of biologically relevant analytes greatly increased in the last decade, owing to the development and application of new macrocyclic receptors [1,2,3,4,5]. Supramolecular host–guest chemistry sets on macrocyclic compounds and uses noncovalent interactions, such as hydrogen bonding, electrostatic forces and π-effects, and is the basis of molecular recognition and sensing. These processes can be monitored by different analytical techniques, but fluorescence spectroscopy has several advantages, such as high sensitivity, simplicity, low cost and real-time detection. In addition, fluorescence-based probes enable in situ noninvasive sensing of biological samples and allow the detection of the analytes inside living cells (e.g., confocal fluorescence microscopy).

Calixarenes [6], phenol-based cyclic oligomers, are among the most extensively studied supramolecular hosts, owing to their structural features. They possess pre-organized hydrophobic cavities and well-defined conformations and can be easily functionalized at their upper and lower rims, resulting in a large diversity of derivatives. These compounds are attractive scaffolds to build more complexed systems, able to perform specific supramolecular functions [7]. Among calix[*n*]arenes, the calix[4]arene platform has been the most investigated. Calix[4]arenes adopt four different conformations (cone, partial cone, 1,2-alternate and 1,3-alternate). The cone conformation is the most widely investigated (followed by the 1,3-alternate).

**Scheme 1:**
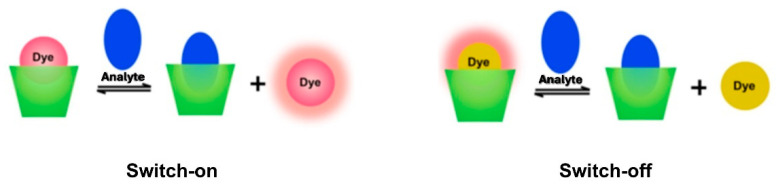
Indicator displacement assay (IDA) for analyte sensing using a macrocycle host (calixarene) and a fluorescent indicator (dye). Adapted from ref. [15].

Lately, a large number of fluorescent sensors based on macrocycles, and in particular, calixarenes, have been used in various applications, namely, in the detection of biologically relevant ions and biomolecules, in bioimaging and in nanomaterials [8,9,10,11]. A calixarene-based fluorescent sensor consists of two components: an ionophore that selectively binds the analyte, forming a host–guest complex, and a fluorophore covalently bound to the calixarene skeleton, which provides a response to signal the binding. According to the situation, this response can be studied by steady-state fluorescence (quenching or enhancement) and/or by time-resolved fluorescence (lifetime constancy or variation) [12]. Calixarenes have intrinsic fluorescence (built-in fluorophore) [13], but this emission occurs in the UV region and is not always responsive to analytes. Thus, different fluorophores, such as naphthalene, anthracene, pyrene, dansyl, coumarin, quinoline and naphthalimide, have been covalently linked to the calixarene skeleton, leading to fluorescent probes for the recognition of different types of analytes [8]. Another strategy to build macrocyclic (calixarene) fluorescent sensors uses the *Indicator Displacement Assay* (IDA), which is based exclusively on noncovalent interactions between the calixarene (the host) and the fluorophore (the guest, also called indicator and usually a dye) [10,14]. In the presence of the analyte (a third species), this replaces the fluorophore in the complex. Out of the calixarene cavity, the fluorophore restores its fluorescence (Scheme 1).

This review presents the advances made in calixarene-based fluorescent sensors and their biological applications in the past fifteen years (from 2009 to date). This review is divided into five major sections: fluorescence mechanisms, detection of biologically relevant ions, sensing of biomolecules, living cells and bioimaging, and nanoparticles and nanoaggregates for imaging and drug delivery. Finally, we highlight the conclusions, some limitations and future perspectives for these macrocyclic fluorescent sensors.

## 2. Fluorescence Mechanisms

**Figure 1:**
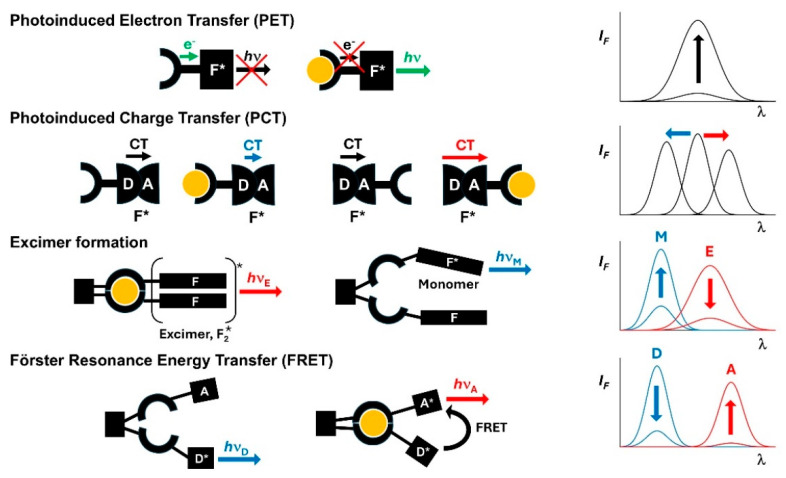
Excited-state processes in fluorescent molecular sensors for ions and molecules. Adapted with permission from ref. [12].

Fluorescence sensing is always based on the change of one or several fluorescence characteristics of a fluorophore (intensity, spectral shift, lifetime, polarization) upon interaction with an analyte (such as a metal cation, an anion or a biomolecule). These changes may simply arise from an alteration of the ground state of the fluorophore upon binding, or they result from a perturbation of an excited-state process by the analyte: electron (or proton) transfer, charge transfer, excimer (or exciplex) formation and resonance energy transfer [12]. These mechanisms, illustrated in Figure 1, are relevant for applications with macrocyclic compounds and are presented in the next sections.

### 2.1. PET (Photoinduced Electron Transfer)

Photoinduced electron transfer can take place from amino groups (electron donors) to excited fluorescent molecules (electron acceptors), e.g., aromatic hydrocarbons, if at close distance. This leads to quenching of their fluorescence [12]. Most fluorescent PET molecular sensors thus consist of a fluorophore linked to an amine moiety via a non-conjugated spacer (e.g., methylene chain). When the amino group interacts with the analyte, it is no longer an effective quencher, and an enhancement of fluorescence occurs. Following protonation (pH indicators) or metal ion binding (ion sensor), the increase is very significant, leading to an *off–on* response.

**Figure 2:**
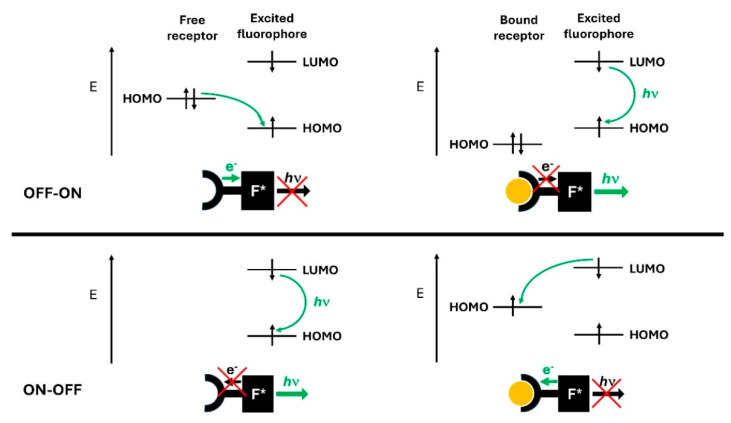
Types of PET fluorescent sensors. Adapted with permission from ref. [12].

The excitation of the fluorophore results in the promotion of an electron in the highest occupied molecular orbital (HOMO) to the lowest unoccupied molecular orbital (LUMO), which enables PET from the HOMO of the donor D (proton-free amine or cation-free receptor) to that of the fluorophore (Figure 2). This results in fluorescence quenching of the latter because the radical anion formed (D^.^^−^) is non-fluorescent. Upon cation binding or protonation, the redox potential of the donor is raised so that the relevant HOMO becomes lower in energy than that of the fluorophore; consequently, PET is no longer possible, and fluorescence quenching does not occur. Conversely, an electron transfer may occur from the fluorophore (electron donor) to a bound analyte (electron acceptor) at close distance, which causes fluorescence quenching (a nonfluorescent radical cation D^.^^+^ being formed). This occurs, for instance, with transition metal ions as analytes. The response is now *on–off* upon binding (Figure 2).

### 2.2. PCT (Photoinduced Charge Transfer)

When a fluorophore contains an electron-donating group (often an amino group) conjugated to an electron-accepting group, excitation often results in significant charge transfer from the donor moiety to the acceptor moiety. This leads to a change in the dipole moment, resulting in a Stokes shift that depends on the micro-environment of the fluorophore. An analyte in close interaction with the donor or the acceptor moiety modifies the intramolecular charge transfer efficiency, thus changing the fluorophore properties [12].

**Figure 3:**
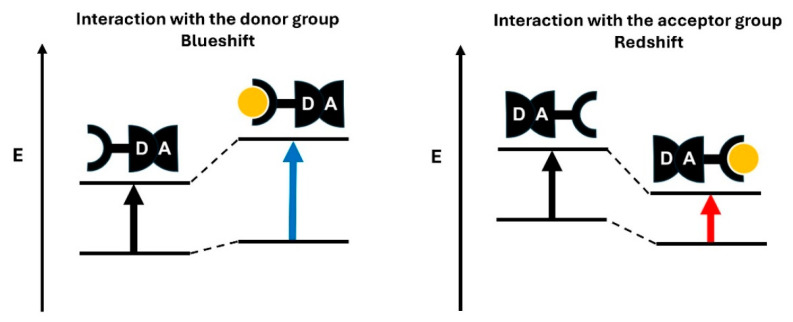
Absorption Spectral shifts in PCT sensors result from the interaction of a bound cation with the electron-donating (**left**) or electron-acceptor group (**right**) of the fluorophore. Adapted with permission from ref. [12].

A cation interacting with the electron donor moiety (e.g., amino) reduces the extent of intramolecular charge transfer. Owing to the resulting reduction in conjugation, a blue-shift of the absorption spectrum occurs, along with a decrease in the molar absorption coefficient (Figure 3). Conversely, a cation interacting with the acceptor group enhances its electron-withdrawing character; hence, the absorption spectrum is red-shifted and the molar absorption coefficient increases. Usually, the fluorescence spectrum shifts in the same direction as the absorption spectrum (Figure 3). These spectral shifts allow for the performance of ratiometric measurements.

In addition to the spectral shift, changes in quantum yield and lifetime are often observed. All the photophysical effects are cation-selective, as they depend on its charge and size.

### 2.3. Excimer Formation or Disappearance

When the analyte binding is accompanied by a change in conformation of the ligand, it is convenient to attach two identical excimer-forming fluorophores in appropriate positions. Two situations are possible [12]. If the fluorophores form an excimer only in the presence of the analyte, owing to favorable conformations brought by its presence, the emission band of the excimer is observed at the expense of the monomer band. Conversely, excimers may already form in the absence of the analyte, but binding of the latter prevents their formation owing to unfavorable conformations. Then the excimer band disappears. In both cases, ratiometric measurements at two different wavelengths (monomer and excimer) are possible.

### 2.4. FRET (Förster Resonance Energy Transfer)

FRET can be used in fluorescence sensing in various ways [12]. When the binding of an analyte induces a major conformational change in a ligand containing both a donor and acceptor, the efficiency of excitation energy transfer is enhanced if the donor–acceptor distance decreases and is reduced if the distance increases. In this way, the relative intensities of the donor and acceptor emissions vary, allowing a ratiometric measurement. Significant changes in intensities are nevertheless observed only if the interchromophoric distance is shorter than the Förster critical radius *R*_0_ in one of the conformations and longer in the other one. Indeed, the transfer efficiency only varies steeply around *R*_0_. The choice of the couple in the donor–acceptor pair, considering the range of relative distances, is thus crucial.

Another possibility is the change in the spectral overlap between the donor fluorescence spectrum and the acceptor absorption spectrum upon binding, which may strongly affect the Förster critical radius *R*_0_.

Finally, the donor and the acceptor may belong to two different entities. The association and the dissociation of these entities will then be accompanied by large changes in transfer efficiencies (on–off behavior in the donor fluorescence).

A recent review discusses FRET in the context of supramolecular macrocyclic chemistry [16].

## 3. Detection of Biologically Relevant Ions

Fluorescent sensors based on calix[4]arenes for the detection of biologically relevant cations have been mainly reported by P. Rao and co-workers [17].

Several lower rim distally substituted triazole-linked calix[4]arene derivatives able to selectively detect the Zn^2+^ cation were investigated by those authors. Fluorescence intensity enhancement was obtained upon addition of Zn^2+^ to receptor **1**, while no significant effect was observed in the presence of the other sixteen interfering metal cations, such as alkali, alkaline-earth, transition and heavy metal [18]. Compound **1** exhibits a detection limit of 36 ppb for Zn^2+^ and an association constant of 1.49 × 10^5^ M^−1^ for the 1:1 complex in aqueous methanolic (1:4) HEPES buffer at pH 7.4. The ability of **1** to sense Zn^2+^ in a biological medium was carried out by fluorescence titrations using blood serum, as well as related albumin proteins (HSA, BSA and α-LA). Almost no changes were observed in the fluorescence intensity of **1** in the presence of these proteins, indicating its utility as a fluorescent turn-on receptor for Zn^2+^.

Rao and co-workers [19] reported the synthesis of other triazole-linked calix[4]arene derivative containing a thiophene unit (**2**) and the high fluorescence emission of the 1:1 complex [**2**∙Zn]. This fluorescent complex was further used to sense molecules containing the thiol (-SH) group by removing Zn^2+^ ion from the complex and forming {Cys/DTT∙Zn} adducts equivalent to those present in metallothioneins. The subsequent release of zinc from the Cys/DTT adduct by heavy metal cations was demonstrated by the recovery of the fluorescence intensity. Thus, calix[4]arene **2** mimics some important steps of the oxidative stress and heavy metal detoxification process present in biological systems.

A zinc complex of the triazole–calix[4]arene derivative **3** was obtained and showed selective recognition to the amino acid cysteine, among several others [20]. Fluorescence titrations were performed under physiological conditions using HEPES buffer, and the complex presented an enhancement of the fluorescence intensity due to a reverse-photoinduced electron transfer process. Significant fluorescence quenching was observed upon addition of Cys, while no response was shown with other amino acids (except His). These fluorescence changes were mainly assigned to the presence of the -SH unit, along with other groups able to de-chelate the Zn^2+^ ion from the complex. This sensing ability of the [**3**∙Zn] complex was tested with other biologically relevant molecules containing the thiol group, and the quenching was obtained followed the order: Cys > GSH > Hcy >> MPA. This study was extended to proteins, such as HSA and BSA, in both their native and reduced forms. The results showed that the [**3**∙Zn] complex was able to differentiate the oxidized (-S-S-) from the reduced (-SH) forms of cysteine containing proteins by the recognition of their cysteinyl units. In addition, the [**3**∙Zn] complex detected free biological thiol contents in reduced blood serum samples. This complex may therefore be used as a receptor for the recognition in vitro of cysteinyl units.


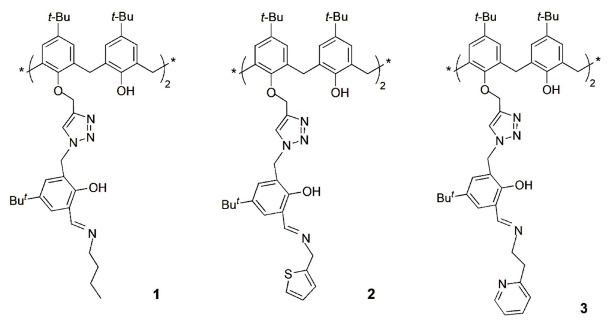


A phenol appended triazole–calix[4]arene derivative **4** was synthesized and used to prepare in situ transition metal ion complexes, such as Mn^2+^, Fe^2+^, Co^2+^, Ni^2+^, Cu^2+^ and Zn^2+^, which integrate various metalloproteins and metalloenzymes [21]. The binding properties of receptor **4** towards these cations were studied by fluorescence. Titrations with Zn^2+^ showed enhancement of the fluorescence intensity, while quenching was observed with the other ions. These [**4**∙M_2_] complexes were able to sensitively and selectively detect the amino acids Glu, Asp, His and Cys. For example, [**4**∙Cu_2_] senses only Cys, while [**4**∙Zn_2_] shows selectivity for His. The results obtained exhibited significant analogies with the biological systems. Compound **4** acts as a multi chemosensor, being the recognition of the biologically active amino acids obtained by the presence of a specific metal ion.

A triazole biscalix[4]arene derivative **5**, bearing a triethyleneglycol di-imine, and its Zn^2+^ complex were obtained, and the [**5**∙Zn_2_] complex was investigated as a chemosensor towards several anions [22]. This highly fluorescent complex showed selective recognition for species containing phosphate, in particular to adenosine triphosphate (ATP^2−^). The fluorescence intensity of [**5**∙Zn_2_] was gradually quenched in the presence of ATP^2−^, reaching a complete quenching after 4–5 equivalents due to its strong binding towards Zn^2+^. The displacement of the cation from the complex was also supported by ESI-MS spectra through the peaks at m/z = 575 and 1309, corresponding to the {Zn∙ATP} adduct and free **5**, respectively. Later on, Rao and co-workers obtained the X-ray crystal structure of the previous complex and used it to sense naturally occurring amino acids [23]. The selectivity towards Cys was the greatest, following by His (97% and 38% fluorescence quenching, respectively), among the amino acids studied. These results indicate the involvement of the thiol group of Cys in the removal of zinc from its complex.


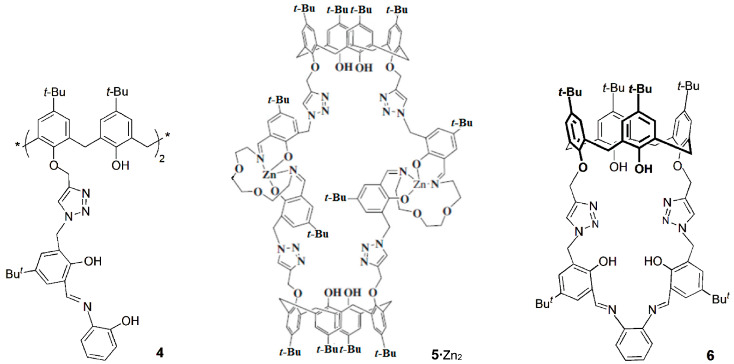


A selective receptor for the Mg^2+^ cation was also reported by Rao and co-workers [24]. The binding behavior of phenylene-diimine capped triazole calix[4]arene derivative **6** was investigated towards the biologically relevant alkali and alkaline-earth ions, Na^+^, K^+^, Mg^2+^ and Ca^2+^. The titration of **6** with Mg^2+^ causes an increased fluorescence intensity of ~70-fold, while no significant enhancement of the fluorescence was observed with the other cations. This receptor can also recognize Mg^2+^ in blood serum milieu, with a detection limit of 0.21 ppm (8.7 μM), and the presence of large amounts of the other ions does not affect its selectivity to Mg^2+^.

**Scheme 2:**
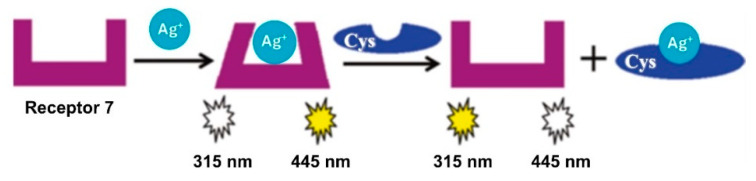
Schematic representation of primary and secondary sensing properties of receptor **7**. Adapted with permission from ref. [25]. Copyright 2009, American Chemical Society.

The lower rim 1,3-diamide-bis(2-picolyl) calix[4]arene derivative **7** showed, by fluorescence studies, high selectivity toward Ag^+^, among several other biologically relevant metal ions [25]. The interaction of Ag^+^ with **7** yielded fluorescence quenching and enhancement at 315 nm and 445 nm, respectively, showing a ratiometric behavior. The [**7**∙Ag^+^] complex prepared in situ recognized Cys, among several naturally occurring aminoacids, by releasing **7** from the complex and forming a new [Cys∙Ag^+^] complex. Therefore, **7** can be considered a potential primary sensor for Ag^+^ and a secondary sensor for Cys (Scheme 2).

The synthesis of fluorescent distally disubstituted calix[4]arene-bearing coumarin-triazole moieties at the lower rim (**8**) was described, as well as its high selectivity for Cu^2+^ ions over other metal cations [26]. Chemosensor **8** showed an appreciable fluorescent quenching upon the addition of Cu^2+^, with a detection limit of 5.4 × 10^−7^ M. Furthermore, **8** forms a 1:1 complex with Cu^2+^, and the sensing mechanism may be due to the reversal of PET and/or the heavy atom effect. The detection of the Cu^2+^ ion was successfully carried out by fluorescence titrations in human blood serum, with 90–100% recovery, indicating that chemosensor **8** could be used as an efficient sensor for Cu^2+^ in real samples.

Relevant anion sensing by fluorescent calixarenes has also been reported [8].

Sutariya and co-workers reported the synthesis of a calix[4]arene quinoline-diamido derivative **9** and its highly selective recognition of Cu^2+^ and F^−^ ions in the presence of other competitive ions [27]. The enhancement or quenching of the fluorescence intensity were observed upon addition of Cu^2+^ or F^−^, respectively, to **9** through PET from the free receptor to the guest. The detection limits of this fluoroionophore were 4.2 nM for Cu^2+^ and 2.2 nM for F^−^, and it was applied to detect Cu^2+^ in blood serum and F^−^ in wastewater, with good results.


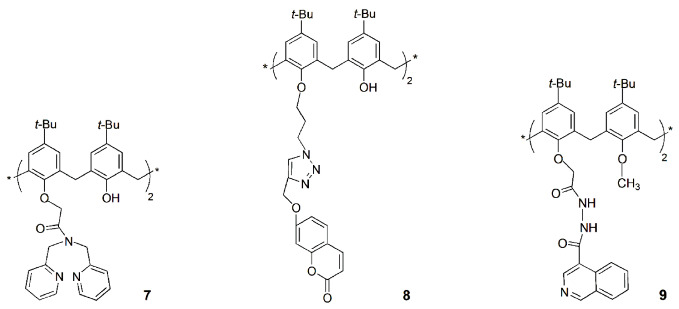


**Figure 4:**
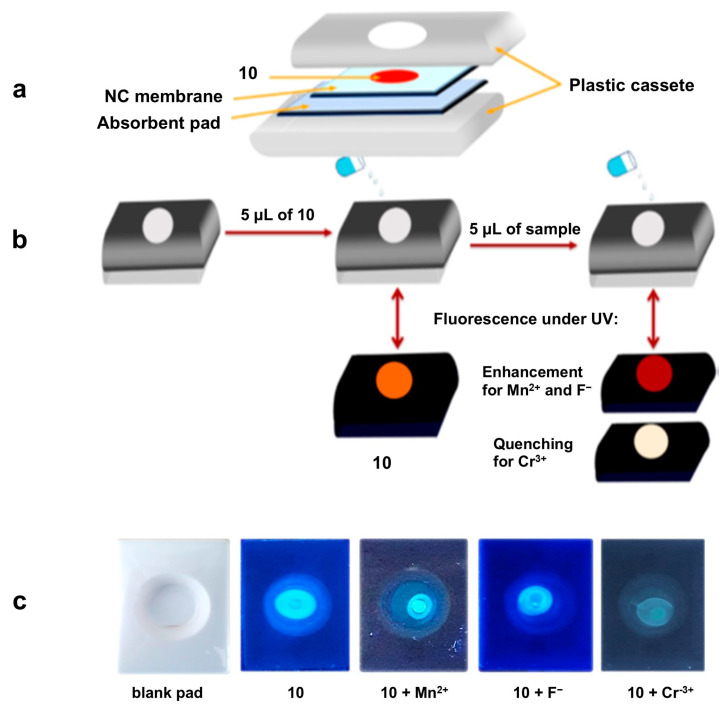
(**a**) Schematic representation of plastic cassette preparation for paper-based analytical device (PAD); (**b**) Stepwise procedure of PAD in presence of sensor **10** and of ions Mn^2+^, Cr^3+^ and F^−^; (**c**) Their response under UV light. Adapted with permission from ref. [28]. Copyright 2019, Elsevier.

Some years later, the same authors obtained a series of calix[4]arene tetra- (**10**) and di-substituted (**11**) derivatives with anthraquinone-amido moieties at the lower rim [28]. The tetra-substituted derivative presents a turn on–off–on type fluorescent probe for Mn^2+^, Cr^3+^ and F^−^ via chelation-enhanced fluorescence photoinduced electron transfer (CHEF-PET) mechanism. It was observed that Mn^2+^ and F^−^ ions enhanced the sensor fluorescence intensity by the CHEF mechanism, while Cr^3+^ quenched it by PET from the free receptor to the guest. Sensor **10** presents limits of detection of 11 nM for Mn^2+^, 4.0 nM for Cr^3+^ and 19 nM for F^−^. This probe was used for the detection of Mn^2+^ in blood serum samples and of Cr^3+^ and F^−^ in industrial wastewaters with 94–99% recovery. In addition, the authors reported an easy-to-use, low cost and replaceable paper-based sensing device for fast analysis of those ions. This device contains the fluorescent sensor immobilized into a thin, porous nitrocellulose (NC) paper membrane, as shown in Figure 4.

Similar studies were conducted with di-substituted calix[4]arene **11** [29]. A selective and sensitive CHEF-PET fluorescent probe based on **11** was obtained for Cu^2+^, La^3+^ and Br^−^. The titrations showed fluorescence enhancement of the sensor in the presence of La^3+^ and quenching in the presence of Cu^2+^ and Br^−^. The detection limits were 0.88 nM for La^3+^, 0.19 nM for Cu^2+^ and 0.15 nM for Br^−^. This sensor was used to determine these ions in real samples, such as industrial soils for La^3+^, blood serum for Cu^2+^ and industrial wastewaters for Br^−^. Furthermore, a similar paper-based system for rapid screening of those ions was also developed.

A lower rim tetra pyrenyl amide calix[4]arene sensor (**12**) was also developed, which showed a very strong interaction with Zn^2+^, Hg^2+^ and I^−^ ions through the CHEF-PET mechanism [30]. This fluorescent probe displays detection limits of 6.4 nM for Mn^2+^, 2.9 nM for Hg^2+^ and 21 nM for I^−^, and binding constants (determined by fluorescence titrations) in the order of 10^8^ M^−1^ for the three ions. The applicability of this probe was tested to sense Hg^2+^ in industrial wastewaters and in salt samples to detect I^−^. Finally, a strip-paper-based sensing device was developed: the addition of different concentrations of Hg^2+^ (from 10^−7^ to 10^−5^ M) displays a fluorescence quenching, while a fluorescence enhancement was observed in the cases of Zn^2+^ and I^−^.


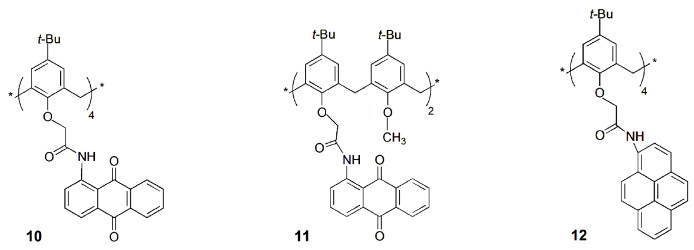


A series of tri-iodotriazole-linked calix[6]arenes (**13**) were studied as transmembrane transporters for Cl^−^ and NO_3_^−^ anions, and their behavior was compared to triazole-linked calix[6]arene **14** [31]. The receptors were incorporated in the lipid bilayer of vesicles containing lucigenin dye (LCG) in a NaNO_3_ solution. The Cl^−^ influx was assessed by fluorescence quenching of the dye. Among receptors, **13a** showed a remarkable selectivity and a good activity (EC_50_~0.007 mol%). This study demonstrated that calixarenes containing halogen bond donor groups are as efficient as others with hydrogen bond donors, showing even a better Cl^−^ selectivity.

The detection limits of various fluorescent sensors for the respective guests are summarized in Table 1.


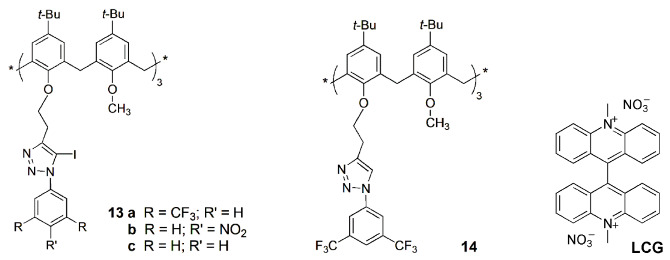


## 4. Sensing of Biomolecules

The recognition of aminoacids, peptides, proteins and other relevant biomolecules by calixarenes able to detect spectroscopy signals (such as fluorescence changes) has increasingly been investigated.

Coleman and co-workers [32] described a fluorescence spectroscopy study of the tri-carboxylatomethylene-mono-heptoxycalix[4]arene derivative **15** to bind to the nucleotide binding domain 1 (NBD1) of multidrug resistance protein MRP1. Moreover, this binding is strongly dependent on Mg^2+^ concentration, with the association constant increasing by 2.5 in the presence of the cation (from zero to 7.5 mM).

The affinity of lower rim distal diamido-calix[4]arene derivatives bearing terminal −COOH groups (**16**) towards aminoacids was studied by fluorescence spectroscopy [33]. The selective recognition of Asp and Glu, among the twenty naturally occurring aminoacids, and of the peptides GSH and GSSG, was obtained, mainly with receptor **16c**, which showed fluorescence intensity enhancement upon addition of the guest. These carboxylic rich aminoacids can form specific H-bond interactions between the receptor arms and that of the guest. The studies were also extended to α-helical proteins, such as BSA, HSA and α-lactalbumin (possessing ~14–16% of Asp and Glu residues).


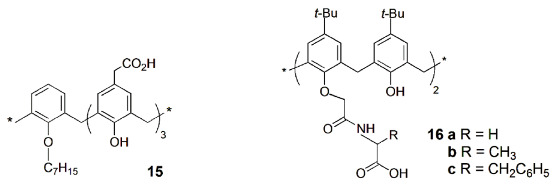


Hof and co-workers reported simple indicator displacement sensors, combining dyes and calixarene host molecules that recognize a wide variety of cationic peptides (histone code analytes, a set of post-translational modifications that control gene expression) [34]. Sulfonated calix[*n*]arenes **17** (*n* = 4, 6) were used together with LCG and MPPE fluorescent dyes. The sensor array components (dye, calixarene, buffer and solvent) are mixed with the analyte, and the signal from each analyte/sensor combination is the fluorescence emission value determined. These sensor arrays operate in homogeneous solutions, can provide continuous data and can read histone code modifications of many types.


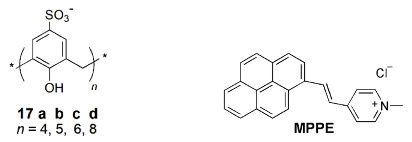


**Scheme 3:**
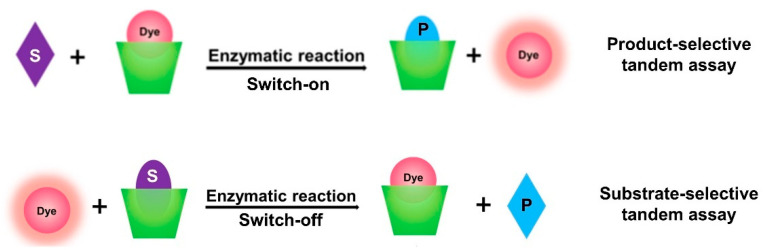
Schematic representation of an enzymatic reaction monitored by tandem assays, using fluorescent dyes and calixarene hosts. S = substrate; P = product. Adapted from ref. [15].

Another important field is the enzymatic activity assays, involving fluorescent supramolecular host–dye receptor pairs, based on the IDA strategy [14,35]. The use of these supramolecular tandem assays to monitor enzymatic reactions has been mainly reported by W. Nau and co-workers. This method combines a suitable fluorescent dye that shows a strong change in fluorescence (quenching or enhancement) upon binding to the host and a macrocycle that differentially binds the substrate and the product by at least a factor of 10 [36]. During the enzymatic reaction, the dye is displaced from the host cavity or taken up into the cavity, resulting in a significant fluorescence response, which can be used to determine the enzymatic activity (see Scheme 3). The water-soluble *p*-sulfonatocalix[*n*]arenes (**17**) have been largely used for such biological applications [1,2].

**Figure 5:**
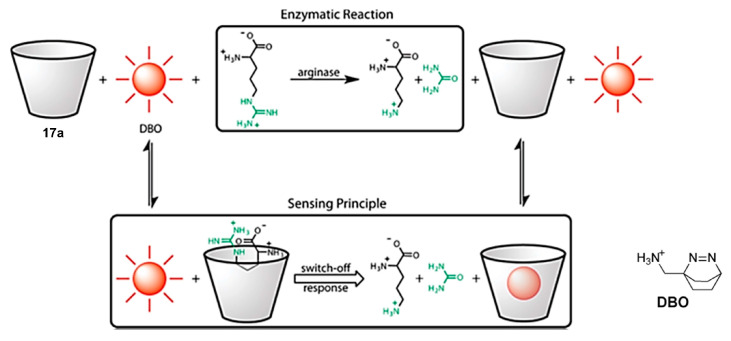
Binding equilibria in a substrate-selective switch-off supramolecular tandem assay for arginase. Adapted with permission from ref. [36] Copyright 2009, American Chemical Society.

In 2009, Nau and co-workers investigated the hydrolysis of arginine to ornithine catalyzed by arginase, using **17a** as the macrocycle and DBO as the fluorescent dye, in which the substrate has a higher affinity to the macrocycle than the product (binding constants of 6.4 × 10^3^ M^−1^ and 5.5 × 10^2^ M^−1^, respectively; substrate-selective assay) [36]. During the enzymatic reaction, arginase converts arginine (stronger competitor) to ornithine and urea (weaker competitors), allowing the dye to form a complex with the macrocycle (binding constant of 6.0 × 10^5^ M^−1^), replacing the substrate. As the fluorescence of the dye is lower in the complexed form, there is a fluorescence quenching of the system, that is, a switch-off response (Figure 5). The authors also used these supramolecular tandem assays to study enzyme inhibition effects.

Water-soluble host–dye reporter pairs composed of **17** (*n* = 4, 5) and LCG were employed by the previous authors to sense acetylcholine and choline, separately and together, in combination with the enzymes acetylcholinesterase and/or choline oxidase [37].

The **17a**∙LCG reporter pair was used to monitor in real-time and in a continuous way the enzymatic trimethylation of lysine residues in peptide substrates by the enzyme lysine methyltransferase [38]. Nau and co-workers encapsulated the previous reporter pair inside liposomes for fluorescence monitoring of the biomembrane transport in real-time [39]. The reporter pair was able to sense protamine (a cationic antimicrobial peptide), heptaarginine (a membrane transduction peptide), the neurotransmitter acetylcholine and the anti-Alzheimer drug amantadine, among other analytes investigated.

Two types of cholinesterase enzymes exist in all vertebrates: acetylcholinesterase (AChE) and butyrylcholinesterase (BChE), and many reported assays are not good enough to conveniently discriminate between them. Liu and co-workers developed a supramolecular tandem assay using the **17a**∙LCG reporter pair to monitor BChE activity [40]. The neuromuscular relaxant succinylcholine was used as the substrate; it can be degraded by BChE, but not by AChE, giving the enzymatic product choline. This system afforded a real-time, continuous and direct sensing of BChE activity through the fluorescence quenching of the dye, being potentially useful in disease diagnosis in which BChE is an important biomarker.

Another reporter pair, combining **17a** and acridine dye (Ac), was used as a fluorescent sensor for the neurotransmitter acetylcholine (AcCh) [41]. Both protonated and neutral forms of the dye were studied at different pH and the results showed a much stronger affinity of the host for the former than for the latter form (binding constants of 1.3 × 10^5^ M^−1^ and 8.1 × 10^2^ M^−1^, respectively), with a sharp decrease in the fluorescence intensity. Upon addition of AcCh to the **17a**∙AcH^+^ complex, a large fluorescence recovery was observed, indicating the displacement of AcH^+^ from the macrocycle cavity by AcCh. Thus, this off–on fluorescent supramolecular system can be potentially applied in the controlled uptake and release of dye/drugs.

More recently, Nau and co-workers reported another supramolecular tandem enzyme assay to monitor phosphatase activity by fluorescence spectroscopy [42]. The reporter pair **17a**∙LCG was used to monitor the phosphorylation of two peptides by kinase proteins. The previous supramolecular reporter pair was employed for the real time detection of saliva pepsin, a biomarker for gastroesophageal reflux disease [43]. Insulin was used as the enzymatic substrate. The displacement of the dye from the **17a**∙LCG complex by the addition of insulin causes the recovery of its fluorescence. After hydrolysis, the lower affinity of aminoacid and peptide products results in regeneration of the **17a**∙LCG complex with fluorescence quenching.


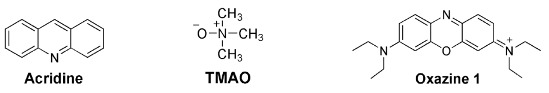


Guo and co-workers reported a supramolecular tandem enzyme assay to evaluate inhibitors of flavin monooxygenase 3 (FMO3), which catalyzes the conversion of trimethylamine (TMA) into trimethylamine *N*-oxide (TMAO), a risk factor in thrombotic diseases [44]. The reporter pair **17a**∙oxazine 1 and the FMO3 catalytic system were used to determine the influence of bioactive compounds with antithrombotic effects from Chinese traditional medicine on that conversion. Receptor **17a** strongly binds the substrate TMA rather than the product (TMAO). The addition of TMA to the **17a**∙oxazine 1 reporter pair leads to an increase in the fluorescence intensity, releasing the dye from the host. This supramolecular approach allowed the successful screening of the bioactive compounds as FMO3 inhibitors.

Calixarenes have been used as surface modifiers of graphene (Gra), leading to hybrid materials that may present new and important biological properties [45]. IDA strategy has also been used in the construction of switch-off–on graphene fluorescent sensing platforms, as graphene behaves as a very good fluorescence quencher, based on the FRET mechanism. C. Li and co-workers developed a fluorescent IDA-based method for drug sensing, combining *p*-sulfonated calix[*n*]arene functionalized graphene as the host and rhodamine B (RhB) as the fluorescent dye. In the first case, authors described the use of the **17c**-Gra/RhB reporter pair to detect tadalafil, the active component of Cialis, a drug used in erectile dysfunction therapy [46]. The addition of RhB to the host causes quenching of the fluorescence intensity of the dye. However, upon addition of the analyte tadalafil to the **17c**-Gra/RhB complex, RhB molecules were displaced by the analyte, leading to the recovery of its fluorescence, along with a turn-on fluorescent signal. The selectivity of the method toward tadalafil was investigated by studying similar analytes such as sildenafil (Viagra) and vardenafil, as well as common molecules present in human blood (such as glucose, sucrose, NaCl, KCl). Negligible changes in the fluorescence intensity of the **17c**-Gra/RhB complex were observed. Tadalafil was also determined in human serum samples with satisfactory results.


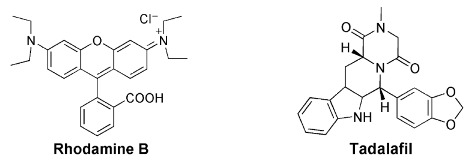


**Figure 6:**
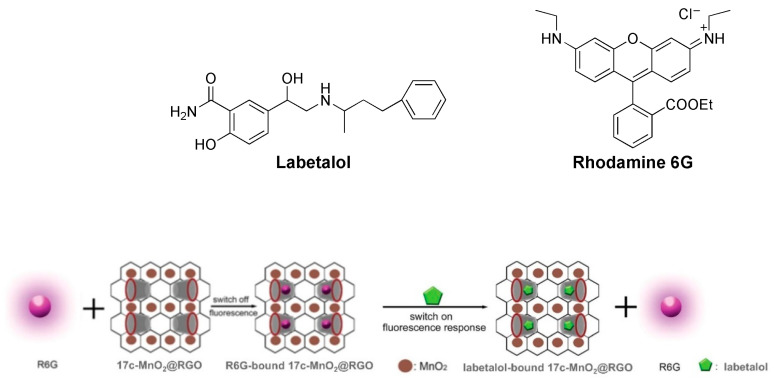
Fluorescent approach for labetalol sensing using R6G/**17c**-MnO_2_@RGO as receptor. Reproduced from ref. [47] with permission from The Royal Society of Chemistry.

One year later, the same authors reported a new fluorescent sensing platform for labetalol determination [47]. Labetalol is an adrenergic β-blocker used in the treatment of hypertension that reduces heartbeat and tremors, being part of the International Olympic Committee list of forbidden substances. In this study, calixarene **17c**, combined with MnO_2_@reduced graphene oxide (**17c**-MnO_2_@RGO), was used as the receptor with rhodamine 6G (R6G) as the dye. When R6G is included in the macrocycle, its fluorescence is quenched by MnO_2_@RGO. It is, however, restored after the addition of labetalol to the **17c**-MnO_2_@RGO/R6G complex, due to displacement of the dye out of the host cavity, therefore, reaching a turn-on fluorescence response (Figure 6). No significant fluorescence changes were observed in the presence of the analogues atenolol and metoprolol. This selective fluorescent probe was also used to determine labetalol in human serum samples.


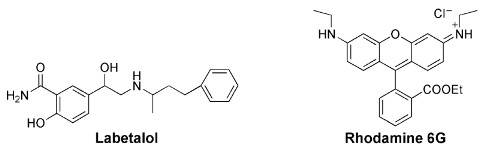


Similarly, in 2016, Li and co-workers reported another study for determination of aconitine (a diester-diterpene alkaloid extracted from some Chinese medicinal herbs) based on a fluorescent sensing platform using **17d**-RGO as the receptor and three different dyes (saframine T, rhodamine B and butylrhodamine B) [48]. The method was successfully applied for the detection of aconitine in human serum samples, as well.

More recently, a metal–organic framework (MOF) fluorescent probe was obtained by the post-synthetic modification of MIL-53-NH_2_(Al) with carboxylatocalix[4]arene (**18**) [49]. The introduction of the calixarene in the MOF improved the fluorescence intensity and the recognition ability of the probe. This fluorescent material was used to sense hippuric acid (HA), showing rapid response, high sensitivity and selectivity and low detection limit (3.7 μg mL^−1^). High levels of hippuric acid in urine can be an indicator of overexposure to toluene. This probe was employed to detect HA in human urine samples, showing potential to be applied as a simple diagnostic method in this area.


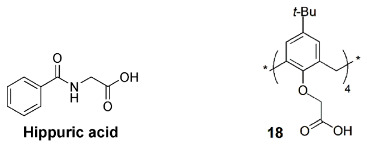


D. Guo and co-workers prepared water-soluble guanidinium-calix[5]arene derivatives bearing alkyl chains at the lower rim (**19**) and have explored them for biomedical applications. Receptor **19a** was used to sense lysophosphatidic acid (LPA), a cancer biomarker, by the IDA mechanism in aqueous media [50]. Fluorescein (FI), the reporter dye, is strongly bound by the host, causing a large fluorescence quenching. The displacement of fluorescein from the **19a**∙FI complex by the addition of LPA results in regeneration of its fluorescence. The sensing of LPA was performed in mouse serum, with a detection limit of 1.7 μM. The tests performed in cancerous and non-cancerous blood samples showed a greater fluorescence response for the former one, indicating potential application of this protocol to diagnose ovarian and other gynecologic cancers in their early stages.


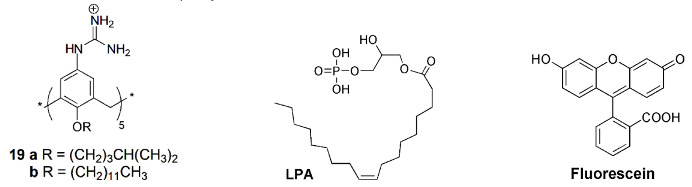


The same authors proposed a new host–guest strategy, named biomarker displacement activation (BDA), for targeted phototheranostics in vivo [51]. The guanidinium-calix[5]arene pentadodecyl ether **19b** was prepared and showed a very strong binding to commercial photosensitizers (PS). They are optical agents that display both fluorescence imaging and photodynamic therapy functions. This strategy was applied in vivo by the construction of a pegylated **19b** nanocarrier. Encapsulation of the photosensitizer in the nanocarrier caused a drastic fluorescence quenching and inhibited its photoactivity. In the presence of an excess of ATP (used as the biomarker) in tumor cells, the photosensitizer is displaced by the biomarker, accompanied by the recovery of the fluorescence and photoactivity. In vivo, studies were carried out using the 4TI tumor-bearing nude mouse model and, comparing with free PS, this **19b**/PS nanosystem was able to selectively visualize the tumor in real time and lead to much more efficient cancer removal. Two years later, these authors applied the same BDA strategy in supramolecular chemotherapy, using the pegylated **19b** carrier and the anticancer drugs oxaliplatin (OX), methotrexate (MTX) and chloroambucil (Chl) [52]. These supramolecular nanodrugs exhibited higher anticancer activities in three cancer cell lines (HeLa, HepG2 and MCF-7) than the corresponding free drugs. The binding affinities of **19b** towards these drugs were determined by fluorescence, via the IDA strategy, using fluorescein as the reporter dye. Fluorescence imaging results clearly showed that **19b** nanocarrier can deliver more drugs into the cells, having a better efficacy than the free drugs.

**Figure 7:**
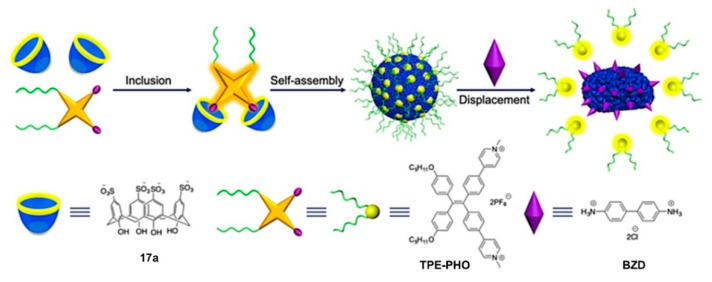
Schematic representation of substitution activated phototheranostics: the dark cytotoxicity and fluorescence of TPE-PHO are inhibited by its complexation with **17a**, but they are recovered by BZD displacement. Adapted with permission from ref. [53]. Copyright 2020, American Chemical Society.

A new substitution strategy based on host–guest interactions to modulate the dark cytotoxicity of photosensitizers (PS) for cancer theranostics was reported [53]. In this system, the PS pyridinium-functionalized tetraphenylethylene (TPE-PHO) showed dark cytotoxicity under light irradiation, but this effect was strongly inhibited after its complexation with *p*-sulfonatocalix[4]arene **17a**. This supramolecular complex emits strong yellow fluorescence in the solution due to the restriction of intramolecular motion of TPE-PHO inside the calixarene cavity. Incubation with HeLa cells showed the TPE-PHO∙**17a** complex mainly located in the cytoplasm. The displacement of PS from the calixarene cavity by the addition of 4,4′-benzidine dihydrochloride (BDZ) translocated it from cytoplasm to mitochondria with high specificity and restores its dark cytotoxicity and photoactivity in the aqueous solution (Figure 7). In vivo, fluorescence imaging studies were carried out with mouse breast cancer cells (4T1), indicating an effective tumor therapy achievement with this system.

The reporter pair **19a**∙fluorescein was used to detect and quantify trimethylamine *N*-oxide (TMAO), a metabolite from intestinal microorganisms, important for disease diagnosis, such as atherosclerosis, thrombosis and colorectal cancer [54]. In this IDA method, the displacement of fluorescein by TMAO from the reporter pair originates a fluorescence enhancement. As TMAO is mostly eliminated from the body through urine, the feasibility of the procedure was tested in artificial and human urine samples. Significant differences in fluorescence were observed between the normal and the normal+TMAO real urine samples.

**Figure 8:**
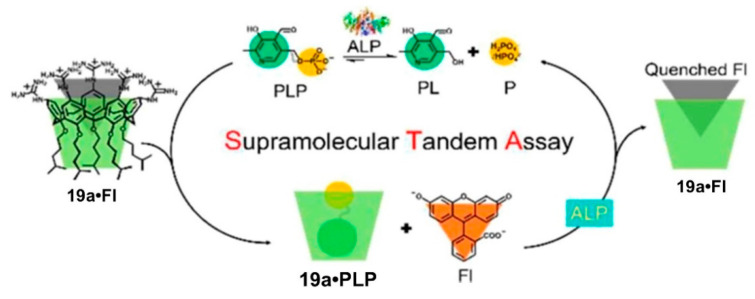
Schematic representation of supramolecular tandem assay built by **19a**∙FI reporter pair to detect PLP. Reproduced from ref. [55].

Another supramolecular tandem assay was developed based on the previous reporter pair to monitor the activity of pyridoxal-5′-phosphate (PLP), the bioactive form of vitamin B6, a biomarker for several diseases [55]. The hydrolysis reaction of substrate PLP by alkaline phosphatase (ALP) to give pyridoxal (PL) and phosphate (P) products was monitored by the fluorescent change in the **19a**∙fluorescein reporter pair. PLP, a strong competitor, displaces fluorescein from the reporter pair, resulting in an enhancement of the fluorescence intensity. After the reaction, the formed products, weak competitors, allow the regeneration of the **19a**∙fluorescein complex with the consequent fluorescence quenching (Figure 8).

A phenanthroline-imidazole calix[4]arene-based fluorescent sensor (**20**) was reported for the detection of metronidazole (MET), an antibiotic and an antiprotozoal used in the treatment of a wide variety of infections [56]. The fluorescence emission of **20** is selectively quenched in the presence of MET, and the coexisting drugs, cations and anions did not interfere with that quenching. The performance of this probe was tested with a real pharmaceutical pill, and a paper-based sensor was also prepared with success.

An anticancer drug carrier based on a carboxylated azocalix[4]arene (**21**) was reported by Guo and coworkers [57]. Seventeen chemotherapeutic drugs, such as doxorubicin (DOX), epirubicin (EPI) and pirarubicin (THP), were complexed with calixarene **21**, improving their solubility and stability and showing binding constants higher than 10^5^ M^−1^ for most of the cases studied. The fluorescence intensity of DOX (the most studied drug) was completely quenched by **21** due to the PET mechanism. A low cytotoxicity of carrier **21** was found in 4T1 cells after 24 h of incubation. The hypoxia response of **21**∙DOX complex was also studied by confocal laser scanning microscopy, showing that under hypoxic conditions, cells treated with the complex exhibited stronger red fluorescence than those in normoxic conditions, suggesting the release of DOX from the complex. This supramolecular carrier presents the essential prerequisites for a successful drug-delivery system.


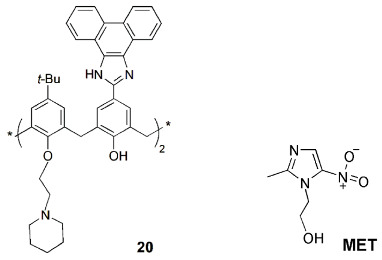


A fluorescence-based method to monitor the membrane transport of peptides in real time was described by Nau, Guo and co-workers [58]. The amphiphilic calix[4]arene **22a** and lucigenin dye form the reporter pair, encapsulated inside the phospholipid vesicles. The binding of peptides to the macrocyclic host leads to the displacement of the dye from the macrocycle, resulting in fluorescence changes that indicate the peptide uptake, confirming its transport through the membrane.


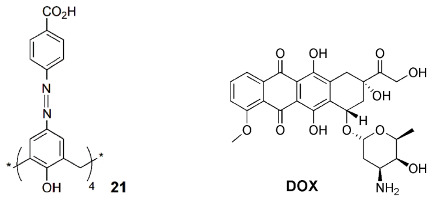


The same authors reported another amphiphilic calix[5]arene (**22b**) as a membrane transport activator of lysine (K)-rich peptides and proteins [59]. Both carboxyfluorescein and lucigenin assays were performed in the liposome transport experiments. Moreover, the uptake of a modified poly K peptide into HepG2 cells (human liver carcinoma) was analyzed by confocal laser scanning microscopy. The cells showed clear fluorescence in the presence of **22b**, indicating the uptake of the peptide even at 4 °C.


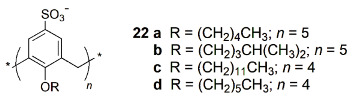


**Figure 9:**
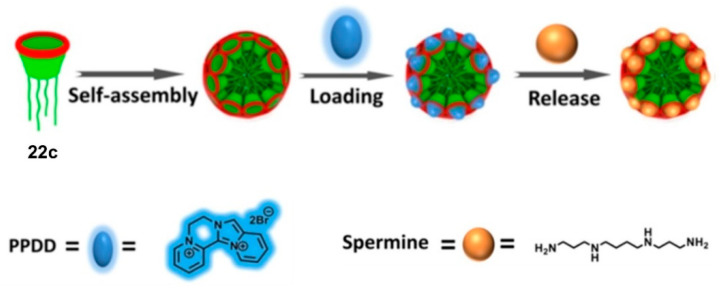
Schematic representation of PPDD ⊃ **22c** for detecting spermine. Adapted with permission from ref. [60]. Copyright 2023, Elsevier.

Very recently, a supramolecular reporter pair composed by an amphiphilic sulfonate calix[4]arene (**22c**) and PPDD was described to detect spermine, a biomarker for cancer diagnosis and treatment, in biological systems [60]. Derivative **22c** showed high binding affinity to PPDD, and its complexation induces fluorescence quenching (Figure 9). In the presence of overexpressed spermine in tumor cells, **22c** can release PPDD, strongly increasing its fluorescence intensity. PPDD ⊃ **22c** was used to detect spermine in HepG2 cells by confocal microscopy, being observed to be a large fluorescence enhancement in the presence of an excess of spermine.

## 5. Studies in Living Cells and Applications in Bioimaging

Due to the important roles that some cations, such as Zn^2+^, Cu^2+^, Fe^2+^ and Al^3+^, have in biological processes, their selective sensing in the presence of several other ions inside the cells is a very demanding task.

Calixarenes have also been used as bioprobes in the cellular medium and as living cell imaging agents. These studies have been conducted by microscopy, both normal fluorescence microscopy and confocal laser scanning microscopy [61]. Thus, the location of these probes based on calixarenes inside the cells can be easily followed due to their fluorescence emission, which results in different colors in the micrographs. Lately, several calixarene-based sensors have been reported in the literature for ion sensing in living cells.

The highly fluorescent [**2**∙Zn] complex (mentioned in Section 3) was also studied in the recognition of phosphate anions in general, and pyrophosphate (PPi) in particular, in HEPES buffer solution [62]. Its fluorescence was strongly quenched in the presence of the P_2_O_7_^−4^ anion, clearly indicating that this complex is a sensitive and selective chemosensor for that anion. Fluorescence microscopy studies were performing using human cervical carcinoma (HeLa cells) to demonstrate the practical utility of [**2**∙Zn] complex. HeLa cells showed very low intracellular fluorescence after incubation with **2**, but upon addition of Zn^2+^, they exhibited intense blue fluorescence. However, when HeLa cells were treated with PPi, fluorescence, quenching was observed from microscopy images. These cellular studies indicated that **2** has good cell permeability and effective intracellular fluorescence emission, forming an in situ complex with Zn^2+^, whose fluorescence intensity decreases upon addition of PPi. The [**2**∙Zn] complex also showed a selective recognition towards His and Cys in HEPES buffer solution, exhibiting a fluorescence quenching only in the presence of these two amino acids, among several others [63]. Cellular studies were also performed using HeLa cells and indicated that the [**2**∙Zn] complex has potential to be a bio-imaging fluorescence probe for His and Cys.

Another zinc complex based on the pyridyl-imino-phenolic-calix[4]arene derivative **3** was shown to be selective to phosphate containing ions and molecules, in particular PPi and ATP [64]. Moreover, derivative **3** is an effective intracellular Zn^2+^ imaging agent with cell permeability. When HeLa cells were incubated with **3**, non-fluorescent images were observed; however, strong fluorescence was obtained in the cells upon Zn^2+^ binding.

**Figure 10:**
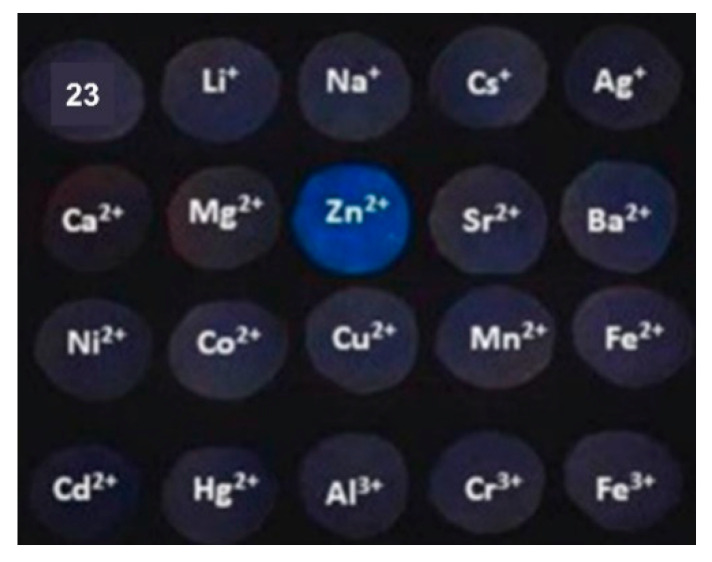
Photographs of the test paper of **23** with different cations in aqueous solution. Reproduced with permission from ref. [65]. Copyright 2019, Wiley.

Erdemir and coworkers synthesized an assembled fluorescent sensor based on a diamido-calix[4]arene and phenolphthalein (**23**) that selectively recognizes Zn^2+^ in aqueous samples and responds to the cation in a reversible way in the presence of Cys, which certifies it for practical applications [65]. Sensor **23** showed a very strong fluorescence enhancement upon addition of Zn^2+^ due to the suppression of the PET process and restriction of C=N isomerization. Moreover, **23** exhibited a detection limit of 0.11 µM for Zn^2+^ and an association constant of 4.5 × 10^11^ M^−2^ for the 1:2 complex in MeCN/H_2_O (8:2). A fluorescent test paper was produced for the easy visualization of Zn^2+^ in aqueous solution (Figure 10). Moreover, fluorescence imaging experiments in human colon cancer cells (DLD-1) indicated that probe **23** can be used to monitor Zn^2+^ in living cells.


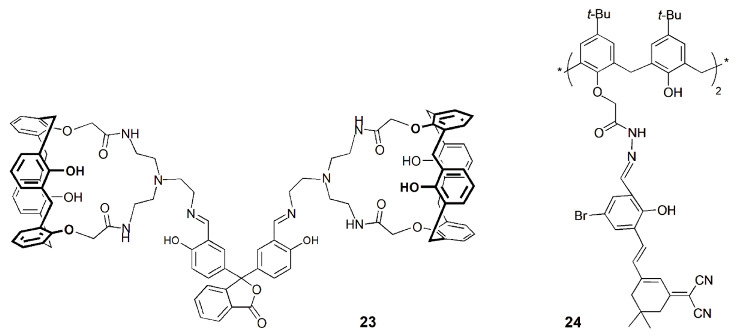


One year later, the same authors obtained a calix[4]arene derivative bearing two isophorone moieties through a 4-bromophenol linker at the lower rim (**24**) and reported its fluorescence sensing for Zn^2+^ [66]. Sensor **24** exhibited selective turn-on fluorometric detection and near-infrared emission for Zn^2+^ over a series of relevant metal ions and a very low detection limit (6.8 nM). Additionally, **24** was used to fabricate a TLC strip sensor for the easy detection of Zn^2+^. Bio-imaging experiments carried out in DLD-1 cells demonstrated that **24** had good cell penetrability and could detect intracellular Zn^2+^ levels.

The synthesis of an upper rim calix[4]arene thiosemicarbazone derivative (**25**) was described, as well as its ability to detect Cu^2+^, Hg^2+^ and mainly the selective recognition of Zn^2+^ by turn-on fluorescence changes [67]. This sensor showed about 240-fold fluorescence enhancement in the presence of Zn^2+^, such fluorescence being visible to the naked eye. It was shown that **25** possesses cytotoxicity activity against A549 human lung cancer cells, with an IC_50_ value of 58 µg/mL after 24 h of in vitro treatment. The cellular uptake was also determined by intracellular fluorescence intensity. Green fluorescence was observed in the cells after 12 h of incubation with **25** and exposure to the Zn^2+^ ion, indicating the penetrating ability of calixarene into the cells.


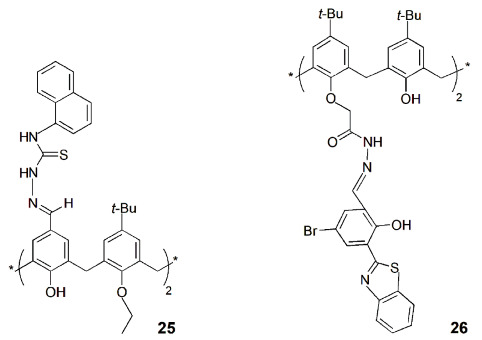


**Figure 11:**
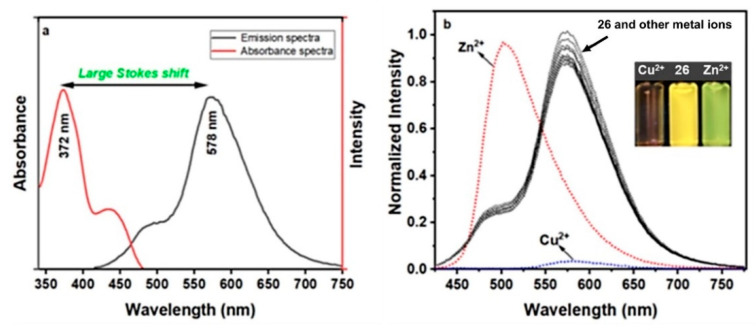
(**a**) Absorption and emission spectra of **26** and (**b**) emission spectrum of **26** (10.0 µM) after the addition of several cations. Adapted with permission from ref. [68]. Copyright 2023, IEEE.

Very recently, Erdemir and coworkers reported a distal-dibenzothiazole-calix[4]arene derivative (**26**) that works as a ratiometric and fluorometric sensor for Zn^2+^ and Cu^2+^ detection [68]. This probe exhibits a very large Stokes shift (206 nm) (Figure 11a) and detection limits of 35 and 66 nM for Zn^2+^ and Cu^2+^, respectively. The addition of Zn^2+^ to an EtOH/PBS buffer solution of **26** causes a color change from yellow fluorescent to green, while the presence of Cu^2+^ originates a strong fluorescence quenching (Figure 11b). This probe can thus selectively discriminate these cations among several others tested. Intracellular imaging studies with probe **26** were performed in HeLa and PNT1 A cells. Negligible cytotoxicity was observed, and the images recorded by the confocal microscope indicated that **26** can monitor and visualize Zn^2+^ and Cu^2+^ in living cells.

The synthesis and fluorescent properties of two azo-calix[4]arene containing anthryl (**27**) or pyrenyl (**28**) moieties at the upper rim were reported [69]. Both derivatives showed Cu^2+^ selectivity by the chelation-enhancement fluorescence process. These compounds were also tested as bio-imaging fluorescent probes to detect Cu^2+^ in the human colon cancer cell line (SW-620) by fluorescence microscopy. Cells incubated with **27** and Cu^2+^ showed clear fluorescence, in opposition to those treated with Cu^2+^ only. Derivative **27** may be used to monitor intracellular Cu^2+^ in living cells.


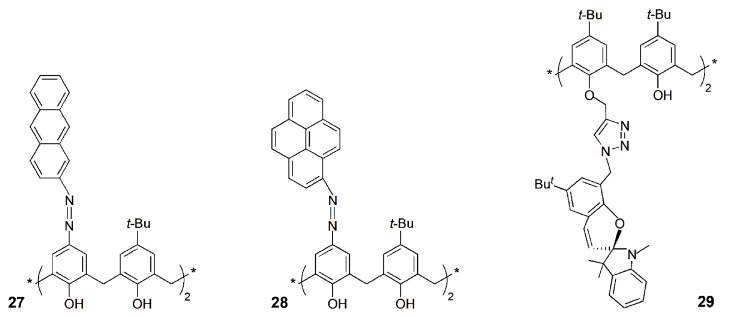


Recently, a lower rim 1,3-di(indoline-spiro-triazole)calix[4]arene derivative (**29**) was synthesized and investigated for its Cu^2+^ binding [70]. The spiro-indoline ring acts as the fluorescent probe and binds Cu^2+^ ion in its open form. Ratiometric fluorescent changes were observed during Cu^2+^ titration, with evidence for the formation of a 1:1 complex and a detection limit of 1.9 nM. Derivative **29** and its Cu^2+^ complex were studied for cancer-cell-killing capacity and also for imaging cancer cells. Only the complex exhibited effective cancer-cell-killing ability with an IC_50_ of 165 nM, suggesting potential to act as an anticancer agent. Cell imaging results showed red fluorescence after incubation of **29** with MDA-MB-231 breast cancer cells, indicating the ability of **29** to accumulate in the mitochondrial region of the cells.

A bis-ruthenium(II) polypyridyl-triazole calix[4]arene complex (**30**∙Ru_2_) was developed as a luminescent probe for the selective detection of Cu^2+^ [71]. The emission of **30**∙Ru_2_ was quenched only in the presence of Cu^2+^, among fifteen metal ions studied, due to the PET process from the excited state of the ruthenium moiety to Cu^2+^ ion. Fluorescence of **30**∙Ru_2_∙Cu^2+^ could be reverted upon addition of sulfide anions to the ensemble by a metal displacement method, forming CuS and regenerating **30**∙Ru_2_ complex. This complex showed low toxicity against A549 human lung cancer cells, and it was used in fluorescence imaging studies, showing its ability to detect Cu^2+^ even in the cellular medium.


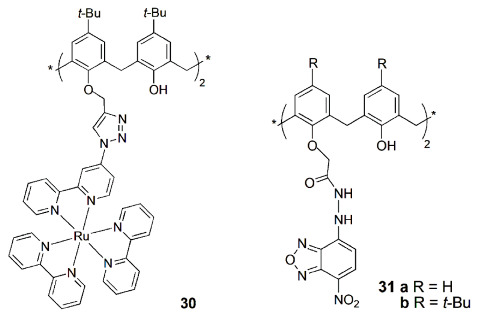


Recently, Yilmaz and coworkers reported the synthesis of two calix[4]arene derivatives bearing nitro-benzofurazan units in 1,3-distal positions at the lower rim (**31a**, **b**) and their ion binding properties [72]. Both sensors showed fluorescence quenching upon the addition of the Cu^2+^ cation, as well as H_2_PO_4_^−^ and F^−^ anions, in a MeCN/H_2_O (4:1) mixture. Confocal microscope imaging was also successfully carried out to identify these ions in A549 cells. A decrease in the intracellular orange fluorescence emission was observed upon complex formation between sensors **31** and those ions.

A selective fluorescent chemosensor for Fe^3+^ over Fe^2+^ based on a quinoline-triazole-linked calix[4]arene derivative (**32**) was reported by Rao and coworkers [73]. Fluorescence imaging studies showed that the [**32**∙Fe^3+^] complex formed in situ enters the cytoplasm of MDA-MB-231 cells and thus can act as an intracellular fluorescent agent.

Rao and coworkers developed a *N*,*N*-dimethylamine ethylimino-triazole linked calix[4]arene derivative (**33**) that showed a turn-on fluorescence upon Cd^2+^ binding [74]. The [**33**∙Cd] complex exhibited high fluorescence emission, selectively recognized Cys, among the twenty naturally occurring amino acids. Fluorescence microscopy studies were carried out using MCF-7 cells and indicated that **33** shows intracellular fluorescence in the presence of Cd^2+^ but loses its fluorescence intensity upon treatment with Cys (switch on and off behavior, respectively).

Later on, the same authors reported a hydroxyethylimino-triazole-calix[4]arene derivative (**34**) and its Cd^2+^ complex [75]. The fluorescence intensity of the [**34**∙Cd] complex is quenched only in the presence of phosphates, mainly H_2_PO_4_^−^, among several anions tested. This is due to the release of **34** from its cadmium complex, through the formation of a ternary species, followed by the capture of Cd^2+^ by the phosphate. Fluorescence microscopy studies were carried out using HeLa cells. These studies indicated that **34** shows cell permeability and effective intracellular fluorescence emission through the formation of an in situ complex, which loses its fluorescence intensity in the presence of H_2_PO_4_^−^.


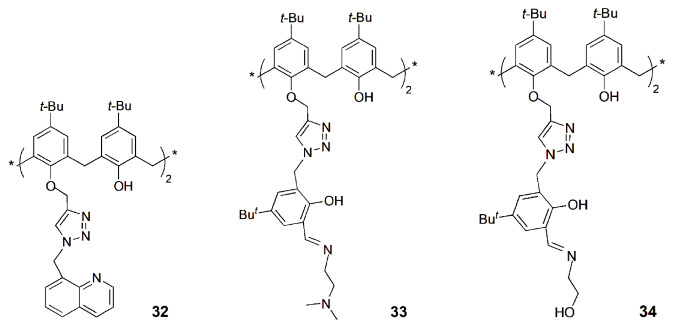


**Figure 12:**
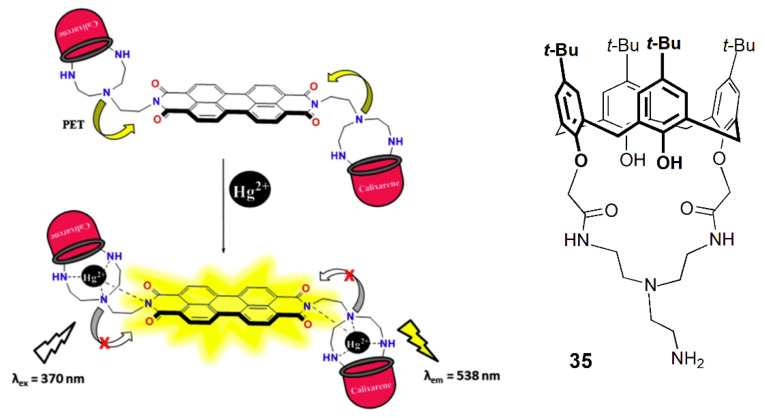
Proposed binding mechanism of perylene bisimide sensor with Hg^2+^ and PET process. Reproduced with permission from ref. [76]. Copyright 2015, Elsevier.

Erdemir and coworkers described a perylene bisimide derivative bearing two calix[4]azacrown units (**35**) as a highly selective fluorescent sensor for Hg^2+^, based on the PET mechanism (Figure 12) [76]. An association constant of 1.6 × 10^9^ M^−2^ in DMF/H_2_O (95:5) was obtained for the 1:2 complex, with a detection limit of 0.56 µM. This perylene-bisimide-based sensor (Figure 12) was used in the fluorescence imaging of human colon cancer cell line SW-620, and the confocal microscopy images obtained indicated that it can be used to detect intracellular Hg^2+^ in living cells.

The synthesis of two water soluble fluorescent *p*-sulfonatocalix[4]arenes bearing dansyl units at the lower rim (**36** and **37**) was described by Yilmaz and coworkers [77]. Fluorescence studies showed that **36** and **37** are selective to Hg^2+^ ion, through switch-off and enhanced PET mechanisms. Compounds **36** and **37** were applied in fluorescence imaging to detect Hg^2+^ in living cells using the SW-620 cell line. The results obtained showed that the nontoxic compounds **36** and **37** can be used to detect intracellular Hg^2+^ in living cells.


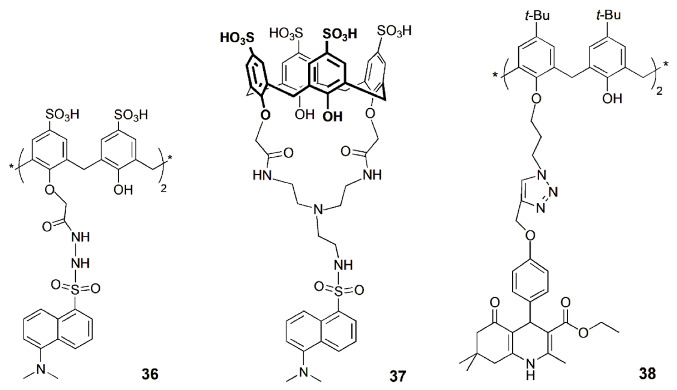


A fluorescent hexahydroquinoline-triazole-linked calix[4]arene derivative (**38**) was described as a selective chemosensor for Hg^2+^ [78]. Fluorescence studies showed a quenching of the emission of **38** upon addition of Hg^2+^, even in the presence of other competitive metal cations, and a detection limit of 0.5 µM. Probe **38** was successfully applied for the detection of Hg^2+^ in real water samples. Moreover, fluorescence microscope images revealed that **38** can easily cross the membrane barrier of lymphocyte cells. Chemosensor **38** did not show any cytotoxic effect on human B cells and was effectively used for Hg^2+^ detection in those cells.


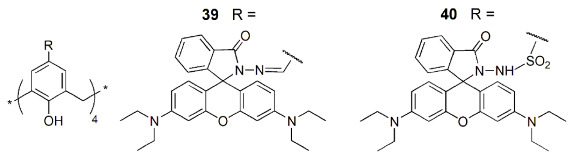


**Figure 13:**
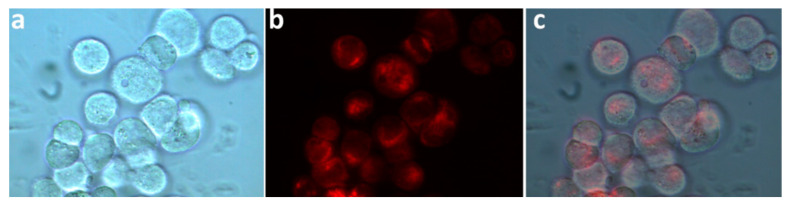
(**a**) Bright-field image of MCF-7 cell incubated with **39** and Hg^2+^, (**b**) fluorescence image of MCF-7 cells incubated with **39** and Hg^2+^, (**c**) merged image of (**a**,**b**). Adapted with permission from ref. [79]. Copyright 2021, Elsevier.

Two other Hg^2+^ sensors based on calix[4]arene derivatives (**39** and **40**) containing four rhodamine units at the upper rim were described [79]. A significant increase in the emission spectra of **39** and **40** was obtained upon the addition of Hg^2+^, while negligible changes were observed in the presence of several other metal ions. Cytotoxicity assay results showed negligible effects on the growth of healthy epithelial cell lines HEK 293 by chemosensors **39** and **40**, but they considerably inhibited the growth of cancer cell lines MCF-7 and MIA-PaCa-2. Fluorescent imaging experiments were also performed, showing that those cells exhibited strong red fluorescence in the intracellular zone only after incubation with both **39** (or **40**) and Hg^2+^ ions (Figure 13). These receptors are thus suitable for real-time detection of Hg^2+^ in living cells, without any damage to healthy cells.


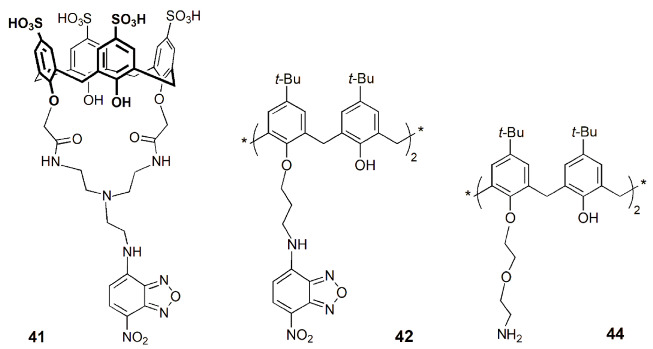


**Figure 14:**
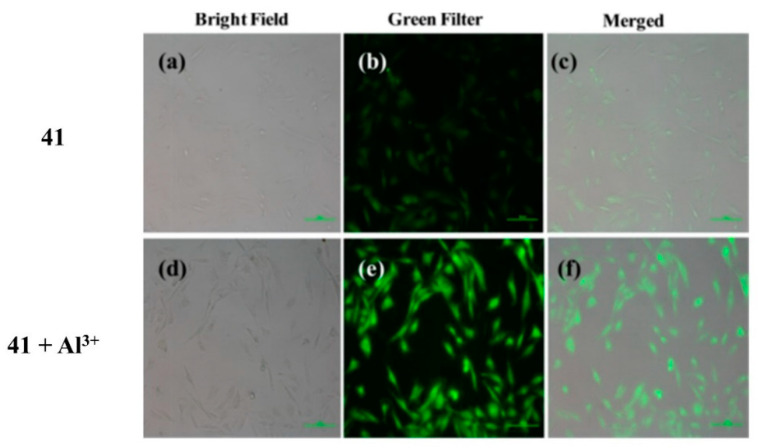
Fluorescence microscopy images of MCF-7 cells: (**a**–**c**) treated with **41** (50 µM); (**d**–**f**) addition of 250 µM of Al^3+^ to **41**. Adapted from ref. [80]. (Open access under an ACS AuthorChoise License).

A nitrobenzooxadiazole-appended calix[4]arene derivative (**41**) bearing a cyclic core connecting the 1,3-positions at the lower rim was developed as a receptor for the selective recognition of relevant trivalent metal cations, such as Cr^3+^, Fe^3+^ and Al^3+^ [80]. A fluorescence enhancement of about 4-fold was observed upon the addition of M^3+^ ions to **41** due to CHEF effect. Binding constants of ~10^4^ M^−1^ and detection limits of 4–5 µM for those M^3+^ ions were obtained, indicating a moderate affinity of **41** for them. Cell imaging studies were also carried out with MCF-7 cell lines by fluorescence microscopy. Weak green fluorescence was observed when the cells were treated with only **41**, but after incubation with Al^3+^ a very bright fluorescence was observed (Figure 14). Thus, **41** is an effective sensor for Al^3+^ in cancer cells.

Another fluorescent lower rim 1,3-dibenzooxadiazole derivative (**42**) was reported by Rao and coworkers as a selective sensor for the fluoride anion [81]. A fluorescence quenching intensity of about 95% was obtained after the addition of F^−^ to sensor **42**, while only marginal changes in its emission spectrum were observed in the presence of all the other seventeen anions studied. Sensor **42** and F^−^ form a 1:1 complex, with a detection limit of 100 nM. Fluorescence and confocal microscopy studies were carried out using HeLa cells. The green fluorescence shown by the cells after incubation with **42** is quenched in the presence of the F^−^ anion and depends on its concentration. Derivative **42** is thus a good sensor for F^−^ in solution and in biological cells.

Consoli and coworkers reported a fluorescent folate-calix[4]arene derivative (**43**), bearing four folic acid units at the upper rim and one nitro-benzofurazan moiety at the lower rim [82]. Confocal fluorescence microscopy experiments were performed to evaluate the cell penetration ability of **43** in normal and cancer cells. The results showed that **43** strongly penetrates inside cancer cells HeLa (>95% of the treated cells showed fluorescence) and A375MM (metastatic melanoma cancer cell line), but exhibited a negligible penetration in normal mouse embryonic fibroblast NIH3T3 cells (~5% of fluorescence). This folate-calixarene derivative selectively enters the cancer cells over the healthy ones by folate-receptor-mediated endocytosis, showing the potentialities of these multifunctionalizable macrocycles in cancer therapy.

Cruz and coworkers described a series of calix[4]arene derivatives bearing amide or amine groups at the lower rim (**44**) and their cellular effects [83]. Fluorescence and confocal fluorescence microscopy studies were carried out to verify the penetration and localization of the calixarenes inside the cells. The results showed that derivative **44** has a remarkable cell anti-proliferative effect and reduces the viability of MCF-7 (breast), LNCaP (prostate), U87 (glioblastoma) and NHDF (fibroblasts) cancer cells even at low micromolar concentrations.

A bifunctional calix[4]arene derivative bearing guanidinium moieties at the upper rim and coumarin units at the lower rim (**45**) was reported by Rao and coworkers to study its DNA binding ability [84]. The transfection ability of **45** was investigated with pCMV-tdTomato-N1 plasmid DNA, which was encoded to synthesize red fluorescent protein in MCF-7 cells, by confocal microscopy. Red and blue (from coumarin unit) fluorescence emission was observed, indicating that DNA was carried inside the cell. Thus, **45** exhibited a good transfection efficiency compared to the commercially available lipofectamine and can be seen as a potential agent to transport genetic material into the cells.



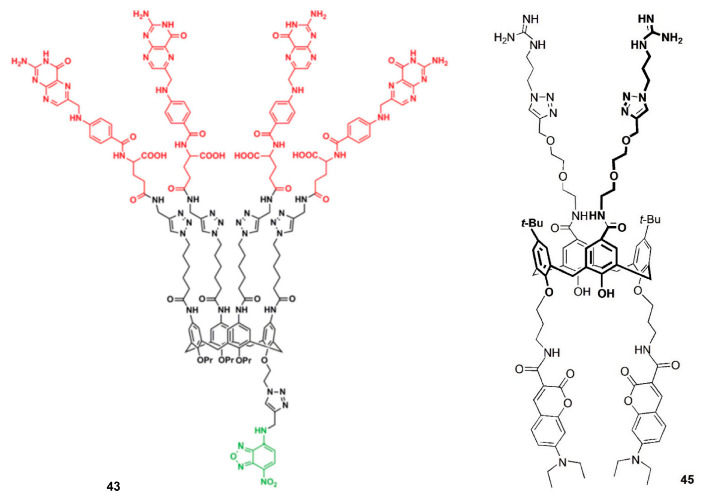

(Reproduced from ref. [82] with permission of The Royal Society of Chemistry.)

The fluorescent water-soluble calix[4]arene **37** was later on used to increase the water solubility and cytotoxicity in cancer cells of naringenin, a flavonoid with important anticancer activity [85]. The [**37**∙naringenin] complex was studied by fluorescence (among other techniques), and a Stern–Volmer constant of 3.5 × 10^7^ M^−1^ was obtained, indicating strong host–guest interaction. The anticarcinogenic effects of this complex were investigated on human colorectal cancer cells (DLD-1) and the results indicated an anti-proliferative effect of DLD-1 cells 3.4-fold more than free naringenin. Fluorescence imaging studies showed that the complex was accumulated into the cytoplasm instead of the nucleus. Calixarene **37** can therefore be a potential candidate for drug carrier.

The detection limits of various sensors and their guests are summarized in Table 2.

## 6. Nanoparticles and Nanoaggregates for Imaging and Drug Delivery

Nanoparticles and nanoaggregates incorporating calixarenes have been used with two main purposes:

(a) the imaging of tissues/cells or specific parts of the cell, e.g., organelles [86,87,88,89,90,91,92] and (b) transport and delivery of drugs to specific targets [93,94,95,96,97,98,99,100].

Water-soluble and amphiphilic calixarenes, which are generally non-cytotoxic, become a key part of the nanoparticle structural scaffold, imparting selective guest capabilities towards specific ionic or molecular hosts, depending on the modifications introduced in these versatile macrocycles. In imaging applications, the guests are luminescent ions or fluorescent organic molecules, either dispersed, complexed or covalently bound.

Jin and coworkers [86] incorporated indocyanine green (ICG), a NIR fluorescent dye approved for medical applications, in small micelles of the hexyl ether derivative of *p*-sulfonatocalix[4]arene (**22d**). The ICG molecule was conjugated to an antibody, allowing the targeting of human breast tumor cells.


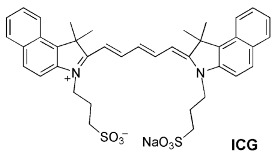


**Figure 15:**
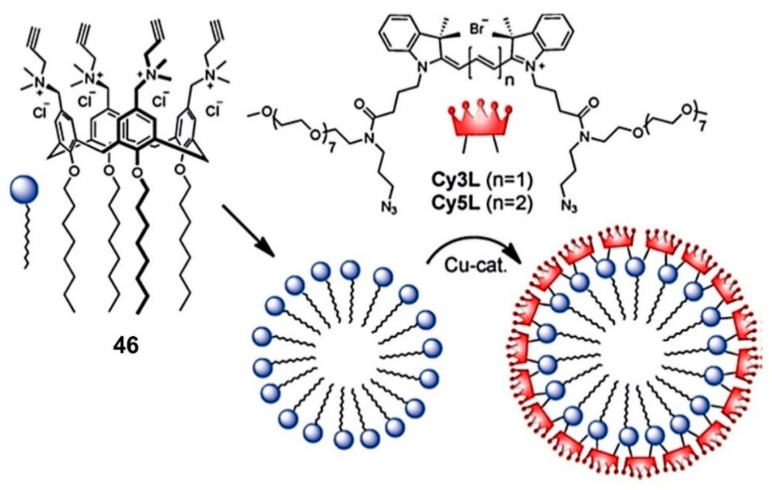
Shell-crosslinking of calix[4]arene **46** micelles, with a cyanine corona. Adapted with permission from ref. [87]. Copyright 2016, Wiley-VCH.

Klymchenko and coworkers [87] developed shell-cross-linked micelles of the amphiphilic calix[4]arene **46**, bearing four alkyne groups at the upper rim, linked to two common fluorescent dyes, cyanines 3 and 5 (Figure 15). Each micelle (7 nm size) contains about 50 fluorophores, has a high brightness and presents very good stability in aqueous and organic media. The applicability of these cross-linked nanoparticles to bioimaging was confirmed with HeLa cells.

The lanthanide complexation capability of modified calixarenes has been used in polyelectrolyte-coated nanoparticles [88]. In these core-shell nanoparticles, calix[4]arene **47** containing tetra-diketone groups at the upper rim complexed both terbium (III) and gadolinium (III). In this way, the particles exhibit both the green emission of Tb(III) and the magnetic properties imparted by Gd(III). These nanoparticles are promising dual magneto-fluorescent bioimaging agents. Optimized Gd-**47**:Tb-**47** ratio (0.2/0.8) of the nanoparticles exhibited a better performance than the commercial Gd(III)-contrast agents.

Amphiphilic nanoparticles formed by the self-assembling of cucurbit[8]uril (**CB[8]**) and dodecyl-linked sulfonatocalix[4]arene **22c**, when loaded with a fluorescent anthracyl pyridinium ionic derivative (ENDT), display a strong red (655 nm) emission and are specific to the lysosome organelle [89]. These nanoparticles were tested for lysosome-targeted imaging in living cells (A549 cancer cells). Only the cells treated with the three component nanoparticles (ENDT + **CB[8]** + **22c**) showed bright red fluorescence in the cytoplasm of the cells.

Micelles of the same calix[4]arene **22c** and NPS (the donor), forming a light-harvesting platform, were used to solubilize a FRET donor–acceptor pair, achieving a strong red (675 nm) emission of the acceptor, Nile Blue [90]. This NIR emissive system maintains its stability and performance in PC-3 cells, mainly in the imaging of the Golgi apparatus.


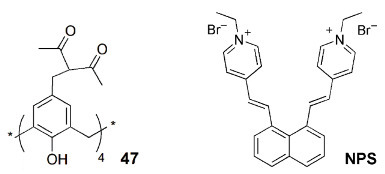


The host–guest complexation capabilities of calixarenes were also used for the enhancement of the fluorescence of aggregation-induced emission (AIE) molecules [91]. When co-assembled with PEG surfactants, calix[5]arene pentadodecyl ether derivative **48** is an effective host for several AIE molecules, giving rise to highly fluorescent water-soluble nanoparticles. These were successfully tested in model systems for image-guided surgery.


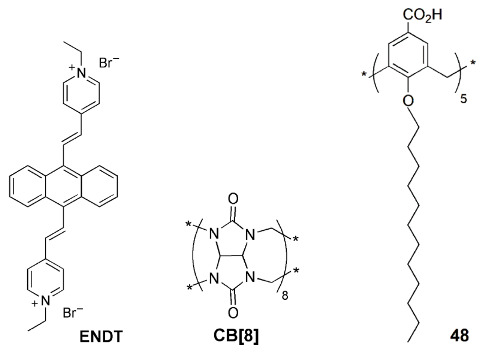


**Figure 16:**
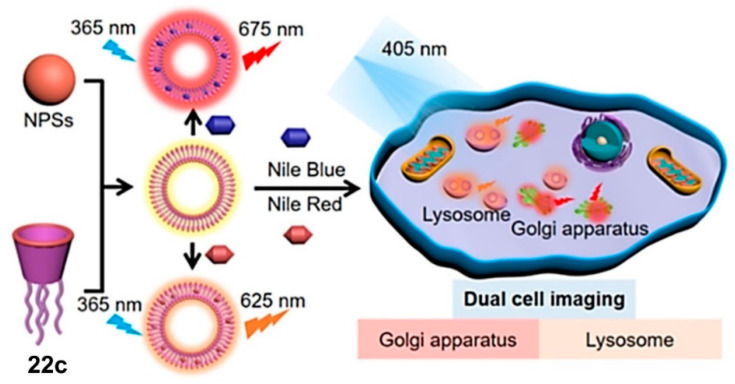
Schematic representation of the AIE nanoparticles. Adapted with permission from ref. [92]. Copyright 2022, American Chemical Society.

Another application of calix[4]arene **22c** is the simultaneous imaging of the Golgi apparatus and of the lysosome with a single excitation wavelength [92]. By incorporating FRET pairs composed of AIE donors (NPSs) and an acceptor dye, strong emission of the acceptor is observed upon donor excitation. Using two sets of particles with the same donors (NPSs) but different acceptors, Nile Red (625 nm emission) and Nile Blue (675 nm emission), and given the different selectivity of these loaded particles with respect to the organelles, it was possible to simultaneously image the Golgi apparatus and the lysosome using the same excitation wavelength (Figure 16).

**Figure 17:**
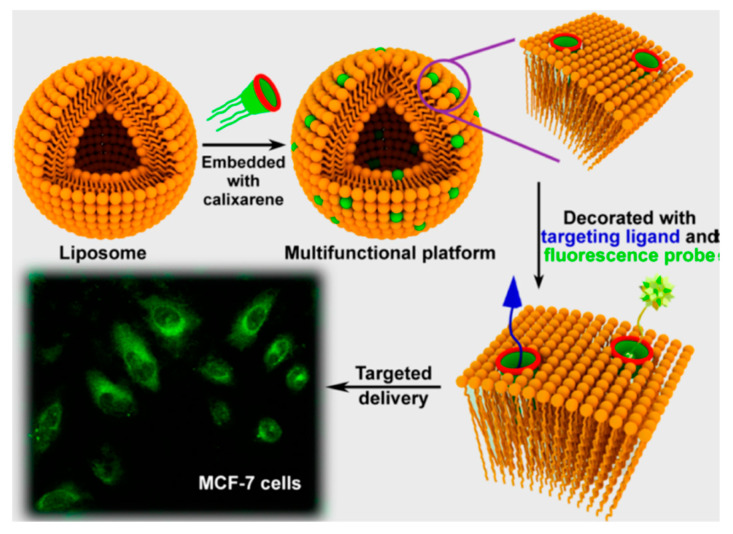
Schematic representation of multifunctional liposome and the noncovalent surface modification via host−guest interaction. Adapted with permission from ref. [94]. Copyright 2015, American Chemical Society.

Calixarenes have also been used in nanodelivery systems carrying specific therapeutic drugs, in most cases aimed at cancer cells. The fluorescence emission of the nanoparticles usually results from a tag fluorophore, but in specific situations, it can come from the drug itself, when intrinsically fluorescent, which is the case of doxorubicin (DOX). Liu and coworkers [93] built binary vesicles using sulfonated calix[4]arene 17a and an asymmetric viologen guest. The vesicles were loaded with DOX, playing the double role of drug and fluorescent reporter. The same authors devised a different class of nanoparticles, by incorporating *p*-sulfonatocalix[4]arenes **22d** and **22e** in natural (phosphoglyceride) liposomes [94]. Decoration of the surface of the particle with biologically active ligands capable of specific targeting gives the nanoparticles the required selectivity (Figure 17). Finally, fluorescence properties were obtained using a non-covalently linked fluorophore.

**Figure 18:**
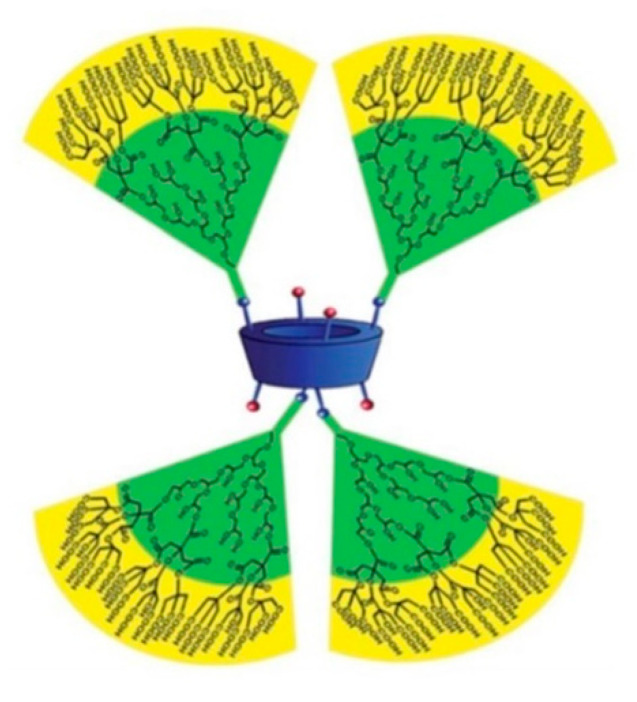
Chemical structure of calix[4]arene **49**-PG-PCA copolymer. Adapted from ref. [95] with permission from The Royal Society of Chemistry.

Nano- and microtubes with a calixarene backbone were obtained by supramolecular assembly of *p*-sulfonatocalix[4]arene **49** in the 1,3-alternate conformation with hydrophilic branching copolymers of polyglycerol and polycitric acid (Figure 18) [95]. The encapsulation of anticancer drug curcumin displayed a strong fluorescence.


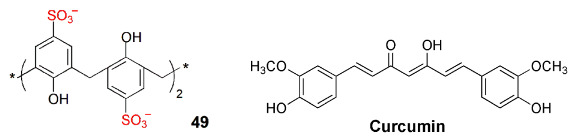


A nano-vehicle based on a lower rim highly branched amine-functionalized *p*-sulfonatocalix[4]arene (**50**) was used as a dual carrier of fluorescent DOX and nonemissive methotrexate (MTX), targeting MCF7 breast cancer cells [96].


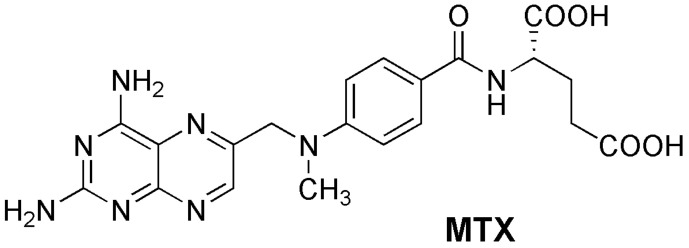
 
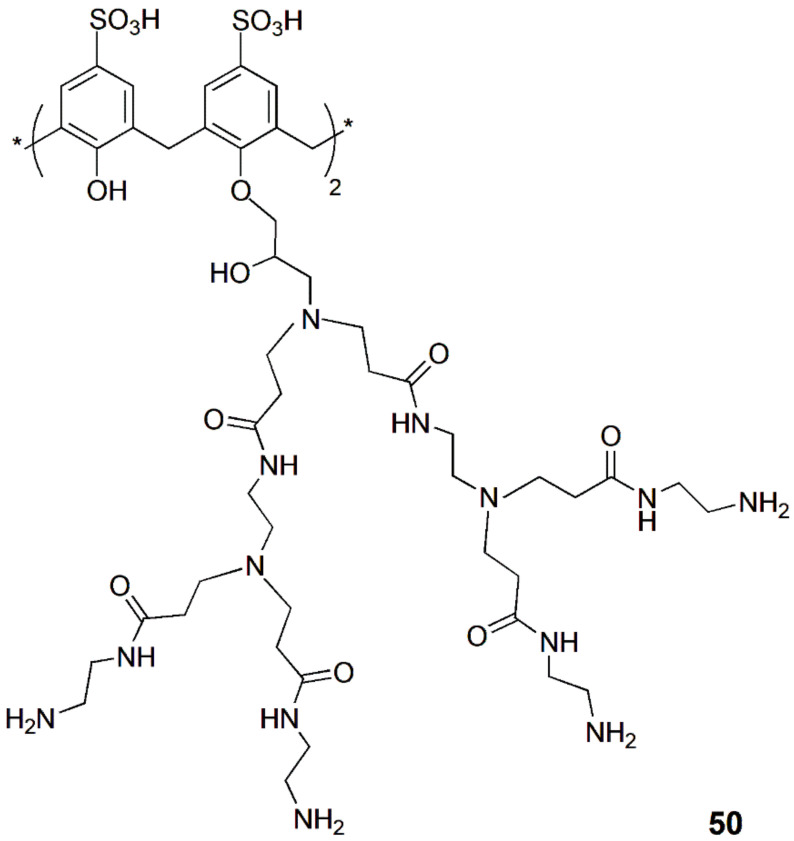


Yang and co-workers [97] designed tumor-specific glucose transporters based on fluorescent labelled calix[4]arene **51**. To the calixarene ring were covalently linked both a monosaccharide and a coumarin fluorophore. In this way, the sugar endows the tumor affinity, whereas the coumarin allows fluorescence tracking.

**Figure 19:**
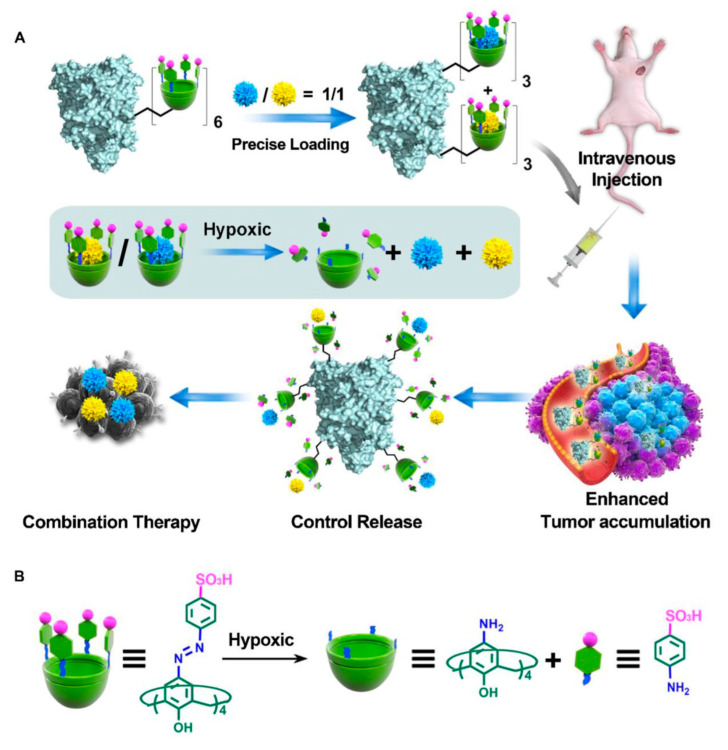
Illustration of calixarene-modified albumin (CaMA) for combination chemotherapy. (**A**) Illustration of CaMA for stoichiometric co-delivery of drug combinations. (**B**) Mechanism of calixarene **52** degradation under hypoxic condition. Reproduced with permission from ref. [100].

Yang Liu and co-workers, in a series of studies [98,99,100], developed further calixarene-containing nanoparticles for cancer therapy. In one of these nanoparticles, therapeutic genes (plasmid DNA) and molecular drugs were combined with no negative interference [98]. This was achieved by complexing the drugs with calix[4]arene **21**, thus preventing direct gene–drug interaction within the carrier (polymer) particles. Evidence was obtained from the measurement of the fluorescence yield of DOX. In subsequent studies [99,100], Liu and co-workers reported nano-drug delivery systems intended to improve the therapeutic performance of drugs while minimizing their side effects. A sulfonated azocalix[4]arene (**52**) was used, and it strongly binds the fluorescent dye Rhodamine B when grafted with poly(hydroxyethylmethacrylate), forming a nonfluorescent complex. However, under hypoxic conditions, it degrades, releasing the dye and restoring the fluorescence. The fluorescence turn-on thus signals the drug release at tumor tissue [99]. This was also shown to occur with bovine serum albumin to which calix[4]arene **52** was surface attached [100], to bind and deliver two different drugs (Figure 19). In this case, one of the drugs was DOX, whose release upon calixarene degradation in hypoxic conditions was again shown by a large fluorescence increase.


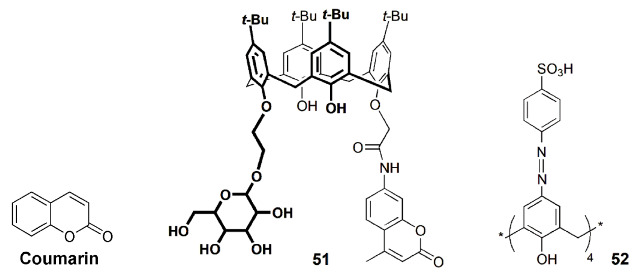


## 7. Outlook

In recent years, a wide variety of calixarenes, in conjunction with many different fluorophores, covalently linked or not, have led to the development of fluorescent systems for numerous applications in Biology and Medicine. This included the detection of biological relevant ions, the selective sensing of biomolecules, such as amino acids, enzymes, drugs and other organic compounds, and also intracellular imaging and drug delivery. Important issues that will need further attention are selectivity, chemical and photochemical stability, and also toxicity when applicable. New water-soluble systems for specific applications not yet covered or up to now only possible in organic solvents seem particularly promising. The broad field of nanoparticles and nanoaggregates for imaging and biomedical applications also offers many still unexplored possibilities.

With respect to the fluorescence methods used, most studies rely on steady-state fluorescence measurements, specifically intensity variations upon binding. Nevertheless, lifetime measurements, in conjunction or not with fluorescence microscopy when appropriate, offer distinctive advantages [12] and allow a better understanding of the interactions involved, and their use to complement the steady-state data is highly recommended.

## Figures and Tables

**Table 1:** Detection limits of several sensors towards various guests.

Sensor	Guest	Detection Limit	Ref.
**1**	Zn^2+^	36 ppb	[18]
**6**	Mg^2+^	0.21 ppm	[24]
**8**	Cu^2+^	5.4 × 10^−7^ M	[26]
**9**	Cu^2+^	4.2 nM	[27]
**9**	F^−^	2.2 nM	[27]
**10**	Mn^2+^	11 nM	[28]
**10**	Cr^3+^	4 nM	[28]
**10**	F^−^	19 nM	[28]
**11**	La^3+^	0.88 nM	[29]
**11**	Cu^2+^	0.19 nM	[29]
**11**	Br^−^	0.15 nM	[29]
**12**	Mn^2+^	6.4 nM	[30]
**12**	Hg^2+^	2.9 nM	[30]
**12**	I^−^	21 nM	[30]

**Table 2:** Detection limits of several sensors towards various guests.

Sensor	Guest	Detection Limit	Ref.
**23**	Zn^2+^	0.11 μM	[65]
**24**	Zn^2+^	6.8 nM	[66]
**26**	Zn^2+^	35 nM	[68]
**26**	Cu^2+^	66 nM	[68]
**29**	Cu^2+^	1.9 nM	[70]
**35**	Hg^2+^	0.56 μM	[76]
**38**	Hg^2+^	0.5 μM	[78]
**41**	Cr^3+^	4–5 μM	[80]
**41**	Fe^3+^	4–5 μM	[80]
**41**	Al^3+^	4–5 μM	[80]
**42**	F^−^	100 nM	[81]

## Abbreviations

AIE	Aggregation-induced emission
Asp	Aspartic acid
ATP	Adenosine triphosphate
BDA	Biomarker displacement activation
BSA	Bovine serum albumin
CHEF	Chelation-enhanced fluorescence
Cys	Cysteine
Cyst	Cystamine
DBO	1-aminomethyl-2,3-diazabicyclo [2.2.2]oct-2-ene
DOX	Doxorubicin
DTT	Dithiothreitol
ENDT	4,4′-Anthracene-9,10-diylbis(ethene-2,1-diyl)bis(1-ethylpyridin-1-ium) bromide
FI	Fluorescein
FRET	Förster resonance energy transfer
Glu	Glutamic acid
GSH	Reduced glutathione
GSSG	Oxidized glutathione
has	Human serum albumin
Hcy	Homocysteine
HEPES	4-(2-hydroxyethyl)-1-piperazine ethane sulfonic acid
His	Histidine
IC_50_	Half-maximal inhibitory concentration
ICG	Indocyanine green
IDA	Indicator displacement assay
α-LA	α-Lactalbumin
LCG	Lucigenin
LPA	Lysophosphatidic acid
MET	Metronidazole
MPA	Mercaptopropionic acid
MPPE	(*E*)-1-(*N*-methylpyridinium-4-yl)-2-(1-pyrenyl)ethene chloride
MTX	Methotrexate
NPSs	Naphthyl-diphenylvinylpiridinium derivatives
PBS	Phosphate buffered saline
PCT	Photoinduced charge transfer
PET	Photoinduced electron transfer
PPDD	Pyrido[1,2-α]pyrido[1′,2′:3,4]imidazo-[2,1-c]-6,7-dihydropyrazinium dibromide
TMAO	Trimethylamine *N*-oxide
